# Ash1l and lnc-Smad3 coordinate *Smad3* locus accessibility to modulate iTreg polarization and T cell autoimmunity

**DOI:** 10.1038/ncomms15818

**Published:** 2017-06-09

**Authors:** Meng Xia, Juan Liu, Shuxun Liu, Kun Chen, Hongyu Lin, Minghong Jiang, Xiaoqing Xu, Yiquan Xue, Wei Liu, Yan Gu, Xiang Zhang, Zhiqing Li, Lin Yi, Youcun Qian, Chen Zhou, Ru Li, Xuan Zhang, Zhanguo Li, Xuetao Cao

**Affiliations:** 1 National Key Laboratory of Medical Molecular Biology, Department of Immunology & Center for Immunotherapy, Institute of Basic Medical Sciences, Peking Union Medical College, Chinese Academy of Medical Sciences, Beijing 100005, China; 2 National Key Laboratory of Medical Immunology & Institute of Immunology, Second Military Medical University, Shanghai 200433, China; 3 Institute of Immunology, Zhejiang University School of Medicine, Hangzhou 310058, China; 4 Institute of Health Sciences, Shanghai Institutes for Biological Sciences, Chinese Academy of Sciences, Shanghai 200025, China; 5 Department of Rheumatology, Peking Union Medical College Hospital, Chinese Academy of Medical Sciences, Beijing 100032, China; 6 Department of Rheumatology & Immunology, Peking University People's Hospital, Beijing 100044, China

## Abstract

Regulatory T (Treg) cells are important for the maintenance of immune homoeostasis and prevention of autoimmune diseases. Epigenetic modifications have been reported to modulate autoimmunity by altering Treg cell fate. Here we show that the H3K4 methyltransferase Ash1l facilitates TGF-β-induced Treg cell polarization *in vitro* and protects mice from T cell-mediated colitis *in vivo*. Ash1l upregulates Smad3 expression by directly targeting *Smad3* promoter to increase local H3K4 trimethylation. Furthermore, we identify an lncRNA, namely lnc-Smad3, which interacts with the histone deacetylase HDAC1 and silences Smad3 transcription. After TGF-β stimulation, activated Smad3 suppresses lnc-Smad3 transcription, thereby recovering the *Smad3* promoter accessibility to Ash1l. By revealing the opposite regulatory functions of Ash1l and lnc-Smad3 in Smad3 expression, our data provide insights for the epigenetic control of Treg cell fate to potentially aid in the development of therapeutic intervention for autoimmune diseases.

## Abstract (web summary)

The transcriptional program activated by Smad2/Smad3 is critical for the induction and function of regulatory T cells. Here the authors show that the expression of Smad3 is modulated by the complementary functions of a methyltransferase Ash1l and an lncRNA lnc-Smad3 on the promoter accessibility of the mouse *Smad3* locus.

Regulatory T (Treg) cells are essential for immune homoeostasis by suppressing effector T cell responses during infection, inflammation and autoimmunity^1^,^2^. Cytokines, particularly transforming growth factor-β (TGF-β) have essential functions for the induction of Foxp3 expression and polarization of Treg cells^3^,^4^,^5^. TGF-β signalling triggers the phosphorylation, activation and nuclear translocation of Smad proteins, such as Smad2 and Smad3, and then the activated Smad complex binds to the *Foxp3* locus and promotes its expression, subsequently leading to Treg cell polarization^6^,^7^. How the TGF-β pathway is regulated to mediate Treg cell development needs further investigation. The mechanisms by which Smad proteins are epigenetically regulated are also poorly understood.

In addition to the well-established functions of transcription factors and cytokines in Treg cell development, other cues, such as epigenetic modifications, are also involved in Treg cell fate^8^,^9^. Previous studies show that Treg cells have a histone H3 lysine 27 (H3K27) trimethylation (H3K27me3) landscape distinct from that of naive T cells and other T helper cells, and appropriate histone modifications induced by activated Smad3 result in the promotion and stabilization of Foxp3 expression^10^,^11^. These studies highlight the involvement of epigenetic modifications in Treg cell development. Characterization of the detailed functions of epigenetic factors in Treg cell-mediated immune tolerance may thus be important for the development of potential interventions for inflammatory autoimmune disease.

Lysine methylation is one of the most characterized histone modifications to date. In particular, H3K4 methylation associated with transcriptional activation is critical for the maintenance of cell fates^12^. In our previous study, the screening of 14 known H3K4 methyltransferases and demethylases for their functions in regulating innate inflammatory immune find ([absent, small, or homeotic]-like [Drosophila]) Ash1l, a H3K4 methyltransferase, as a potent inhibitor of LPS-induced IL-6 production in macrophages^13^. We demonstrate that Ash1l inhibits TLR-triggered innate inflammatory response in macrophages by directly targeting *Tnfaip3* promoter region to induce its expression. Ash1l-silenced mice are more susceptible to bacterial infection and autoimmune diseases^13^.

Ash1l is highly expressed in CD4^+^ T cells^13^. Exogenous expression of the plant homeodomain-like zinc finger domain of Ash1l inhibits the development of CD4^+^CD8^+^ thymocytes *in vitro*, suggesting that Ash1l may regulate early thymic T cell development^14^. In the gut, gene profiling data show that Ash1l is downregulated in colon tissues during T cell-mediated colitis in mice (GSE27302)^15^, and has lower (−2.9-fold) expression in epithelial biopsy samples of inflamed ascending colon than that in uninflamed sigmoid colon of ulcerative colitis patients (GSE11223)^16^. Lastly, *Ash1l* gene also constitutes part of the *Idd17* locus associated with increased susceptibility to autoimmune diabetes in NOD mice^17^. Combined, these data indicate a potential connection between Ash1l and T cell-mediated autoimmune disease.

In this study, we show that Ash1l upregulates Smad3 expression by directly activating its promoter, and thereby promoting Foxp3 expression and induced Treg (iTreg) cell differentiation *in vitro*. By contrast, a newly-identified long non-coding RNA (lncRNA), lnc-Smad3, recruits HDAC1 to the *Smad3* promoter and selectively suppresses Smad3 but not Foxp3 expression. Interestingly, activation of the TGF-β/Smad3 axis suppresses lnc-Smad3 transcription, restoring accessibility of the *Smad3* promoter to Ash1l. Ash1l-silenced mice are more susceptible to T cell-mediated colitis due to the impairment of Treg cell polarization. Lastly, ASH1L, FOXP3 and SMAD3 are downregulated in peripheral CD4^+^ T cells from patients with rheumatoid arthritis. Our results provide insights for the epigenetic control of Treg cell polarization during immune homoeostasis, and suggest a possible association between Ash1l and immune disorders.

## Results

### Ash1l enhances Treg cell polarization by upregulating Foxp3

In our previous study, we already observed preferential expression of Ash1l in CD4^+^ T cells, which inspired us to further analyse the function of Ash1l in T cell development and differentiation^13^. We first analysed the phenotype and proportion of major T cell subsets in Ash1l-silenced mice, which were generated via inserting the PB transposon between exons 15 and 16 of *Ash1l* allele as compared with wild-type (WT) mice^13^. As shown in our previous study, the frequency of CD4^+^ and CD8^+^ T cells in splenocytes from Ash1l-silenced mice was normal^13^. The proportions of CD4^+^ single-positive (CD4SP) and CD8SP thymocytes, activated CD44^hi^CD62L^lo^ CD4^+^ T cells in the spleen and mesenteric lymph nodes (mLN) were also normal in Ash1l-silenced mice (Supplementary Fig. 1a–d). Ash1l-silenced mice also had similar frequencies of Foxp3^+^ Treg in CD4SP thymocytes and peripheral CD4^+^ T cells from spleen and mLN (Supplementary Fig. 1e,f). Thus, silencing of Ash1l does not affect T cell development.

We then cultured naive CD4^+^ T cells from WT or Ash1l-silenced splenocytes under Th1, Th17 or induced Treg (iTreg) cell-skewing conditions to analyse the function of Ash1l in differentiation of CD4^+^ T cell subsets. Notably, we observed significantly impaired iTreg cell differentiation, but increased Th1 and Th17 differentiation in Ash1l-silenced CD4^+^ T cells as compared with WT CD4^+^ T cells (Supplementary Fig. 2a,b). In addition, naive CD4^+^ T cells from Ash1l-silenced mLN or CD4SP thymocytes also showed considerably attenuated induction of iTreg cells (Supplementary Fig. 2c,d). We also found that the expression of Ash1l did not change during iTreg cell differentiation and was also similar among different CD4^+^ T cell subsets, including naive CD4^+^ T cells, Th1, Th17 and iTreg cells (Supplementary Fig. 3a,b), indicating that Ash1l expression in CD4^+^ T cells may not be regulated during iTreg or T helper cell differentiation. In addition, we found that naive CD4^+^ T cells from WT or Ash1l-silenced splenocytes showed similar expression levels of the other H3K4 methyltransferases (Supplementary Fig. 3c), indicating that the deficiency of Ash1l does not affect the expression of other H3K4 methyltransferases.

**Figure 1:**
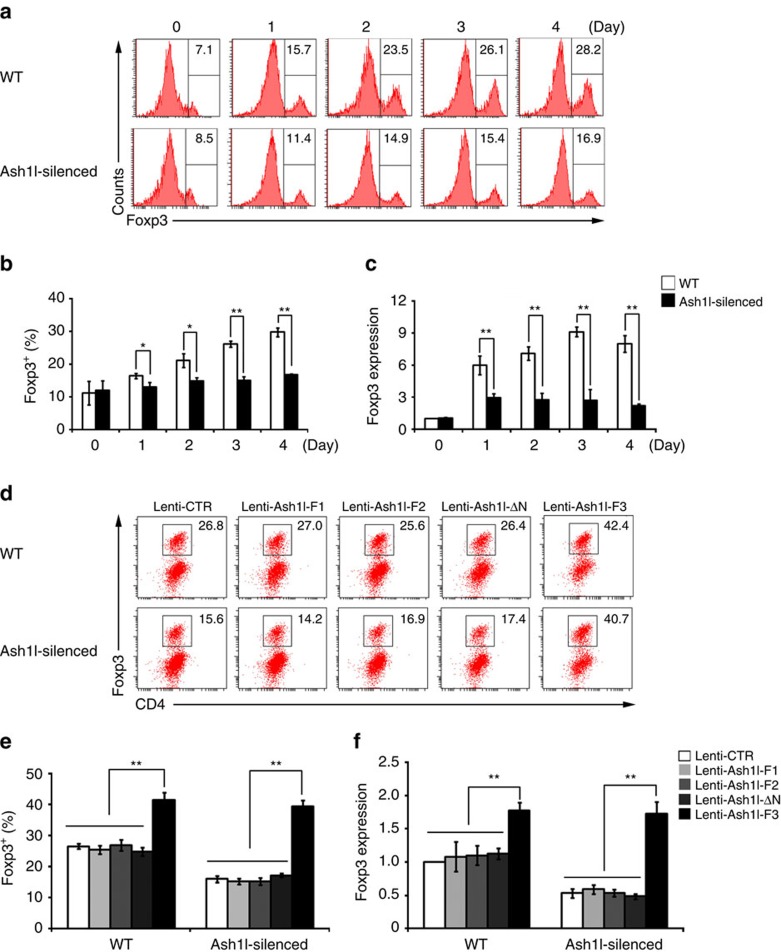
Ash1l facilitates TGF-β-mediated Treg cell induction via promoting Foxp3 expression. (**a**,**b**) Representative flow cytometry (**a**) and quantification (**b**) of the percentage of Foxp3 in the CD4^+^ T cells stimulated under iTreg cell-skewing conditions (with TGF-β) for indicated times. (**c**) mRNA expression of Foxp3 in the WT and Ash1l-silenced CD4^+^ T cells stimulated under iTreg cell-skewing conditions (with TGF-β) for indicated times. (**d**,**e**) Representative flow cytometry (**d**) and quantification (**e**) of the percentage of Foxp3 in WT and Ash1l-silenced CD4^+^ T cells transduced with Ash1l-fragment-expressing lentivirus (Lenti-Ash1l-F1, Lenti-Ash1l-F2, Lenti-Ash1l-F3 or Lenti-Ash1l-ΔN) and cultured under iTreg cell-skewing conditions (with TGF-β) for 3 days. (**f**) mRNA expression of Foxp3 in WT and Ash1l-silenced CD4^+^ T cells transduced and cultured as in **d**. Error bars represent s.d. Student's *t* test. *P<0.05, **P<0.01. Data are representative of three independent experiments (**a**,**d**) or are from three independent experiments (**b**,**c**,**e**,**f**; mean±s.d.).

Since Treg cells are defined by expression of transcriptional factor Foxp3, which is critical for the maintenance and function of Treg cells, we next examined the effect of Ash11 on Foxp3 expression and the function of iTreg. The expression of Foxp3 (Fig. 1a–c) was significantly reduced in Ash1l-silenced splenic CD4^+^ T cells under iTreg cell-skewing conditions (with TGF-β). Accordingly, Ash1l-silenced Foxp3^+^ iTreg cells showed lower suppressive functions, with reduced expression of costimulatory molecules CD25, CTLA-4 and GITR on iTreg cells (Supplementary Fig. 4a) and attenuated capacity of suppressing the proliferation of WT effector T cells (Supplementary Fig. 4b,c). Altogether, these data suggest that Ash1l is critical for TGF-β-mediated induction and activation of iTreg cells by promoting Foxp3 expression *in vitro*.

We next utilized lentivirus-mediated overexpression of Ash1l to confirm the positive effect of Ash1l on the induction of Foxp3 expression and iTreg cells. Considering the nearly 9 kb cDNA length of mouse Ash1l, we constructed Ash1l-fragment 1, 2, 3 (Ash1l-F1, -F2 and -F3) lentiviruses expressing 1–880 amino acids (aa), 881–1,855 aa and 1,886–2,958 aa containing SET (Su[var]3-9, E[z] and trithorax) domain of Ash1l, respectively. We previously showed that the single amino acid point mutation Ash1llΔN (N2212I) had abolished H3K4 methyltransferase activity^13^, so we also constructed Ash1lΔN (N2212I)-expressing lentivirus (Ash1l-ΔN). We observed that only overexpression of lentivirus Ash1l-F3 containing SET domain and normal H3K4 methyltransferase activity could enhance Foxp3 expression and iTreg cell polarization, rescuing the induction of Foxp3 and iTreg cells, which were impaired in Ash1l-silenced CD4^+^ T cells (Fig. 1d–f). Collectively, these data indicate that Ash1l promotes TGF-β-induced Foxp3 expression and iTreg cell polarization dependent on its SET domain-mediated H3K4 methyltransferase activity.

### Ash1l-silenced mice are more susceptible to colitis

**Figure 2:**
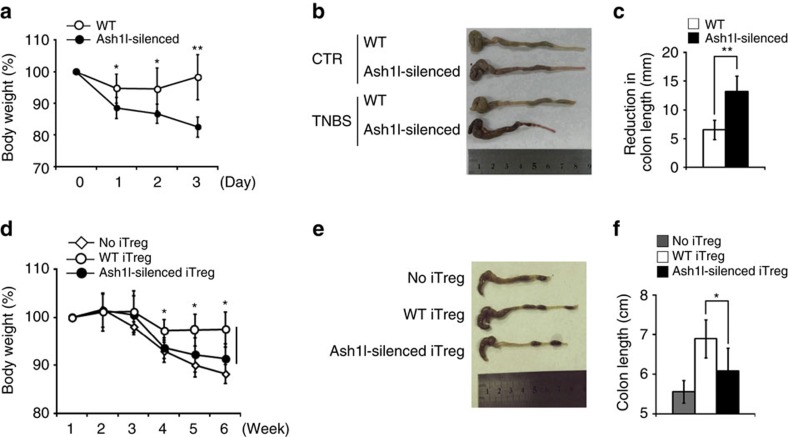
*In vivo* silencing of Ash1l renders mice more susceptible to experimental colitis. (**a**–**c**) TNBS-colitis was induced in 8-week-old WT and Ash1l-silenced mice (*n*=5 mice per group) and monitored for disease development for 3 days after TNBS induction. (**a**) Weight change over time of WT and Ash1l-silenced mice after TNBS induction, presented relative to weight at day 0, set as 100%. (**b**,**c**) Appearances (**b**) of colons, reduction of colon length (**c**) were examined at day3 after TNBS induction. (**d**–**f**) T cell adoptive transfer-mediated colitis was induced in 6-week-old Rag1^−/−^ mice via transferring with WT CD4^+^CD25^−^ T cells with or without WT or Ash1l-silenced iTreg cells (*n*=5 mice per group). Disease development was monitored for 6 weeks. (**d**) Weight change over time of Rag1^−/−^ mice transferred with WT CD4^+^CD25^−^ T cells with or without WT or Ash1l-silenced iTreg cells, presented relative to weight at week 1, set as 100%. (**e**,**f**) Appearances (**e**) and length (**f**) of colons were examined at week 6 after T cell transfer. Error bars represent s.d. Student's *t* test. *P<0.05, **P<0.01. Data are from three independent experiments (**a**,**c**,**d**,**f**; mean±s.d. of five mice) or are representative of three independent experiments (**b**,**e**).

Treg cells are essential for prevention of inflammatory diseases and maintenance of immune homoeostasis. Next, we sought to investigate whether Ash1l was involved in regulation of T cell-mediated autoimmunity *in vivo*. We first investigated the susceptibility of Ash1l-silenced mice to the induction of 2,4,6-trinitrobenzene sulfonic acid (TNBS)-induced colitis. Compared to WT mice, Ash1l-silenced mice developed more severe colitis upon TNBS induction, with a sharper loss of body weight, more exaggerated shortening of colon in appearance, and more severe colon inflammation as indicated by extensive infiltration of mononuclear cells, reduction of goblet cells and mucosa erosion in histology (Fig. 2a–c; Supplementary Fig. 5a). Moreover, upon TNBS induction, Ash1l-silenced mice had fewer Foxp3^+^ Treg cells in the spleen and mLN (Supplementary Fig. 5b), indicating that Ash1l could promote Treg cell differentiation *in vivo* under pathological conditions, which is critical for the prevention of overall inflammatory responses in colitis.

To further confirm whether Ash1l contributes to the prevention of colitis via modulating Treg cell function *in vivo*, we used a T cell adoptive transfer model of chronic colitis^18^. WT CD4^+^CD45RB^hi^ T cells were transferred into Rag1^−/−^ mice together with either WT or Ash1l-silenced *in vitro* differentiated iTreg cells. While WT iTreg cells efficiently protected mice from colitis, Ash1l-silenced iTreg cells failed to inhibit intestinal inflammation and weight loss (Fig. 2d–f; Supplementary Fig. 5c). We also tracked the fate of naive CD4^+^ T cells from WT and Ash1l-silenced mice upon adoptive transfer into Rag1^−/−^ hosts. Compared to WT CD4^+^ T cells, fewer Ash1l-silenced CD4^+^ T cells converted into Foxp3^+^ iTreg cells (Supplementary Fig. 5d,e), which demonstrated that Ash1l promoted iTreg cell polarization *in vivo*.

Altogether, these data indicate that Ash1l prevents the development of T cell-mediated colitis by upregulating Treg cell polarization and function *in vivo*.

### Ash1l enhances Smad2/3-TGF-β signalling via its SET domain

**Figure 3:**
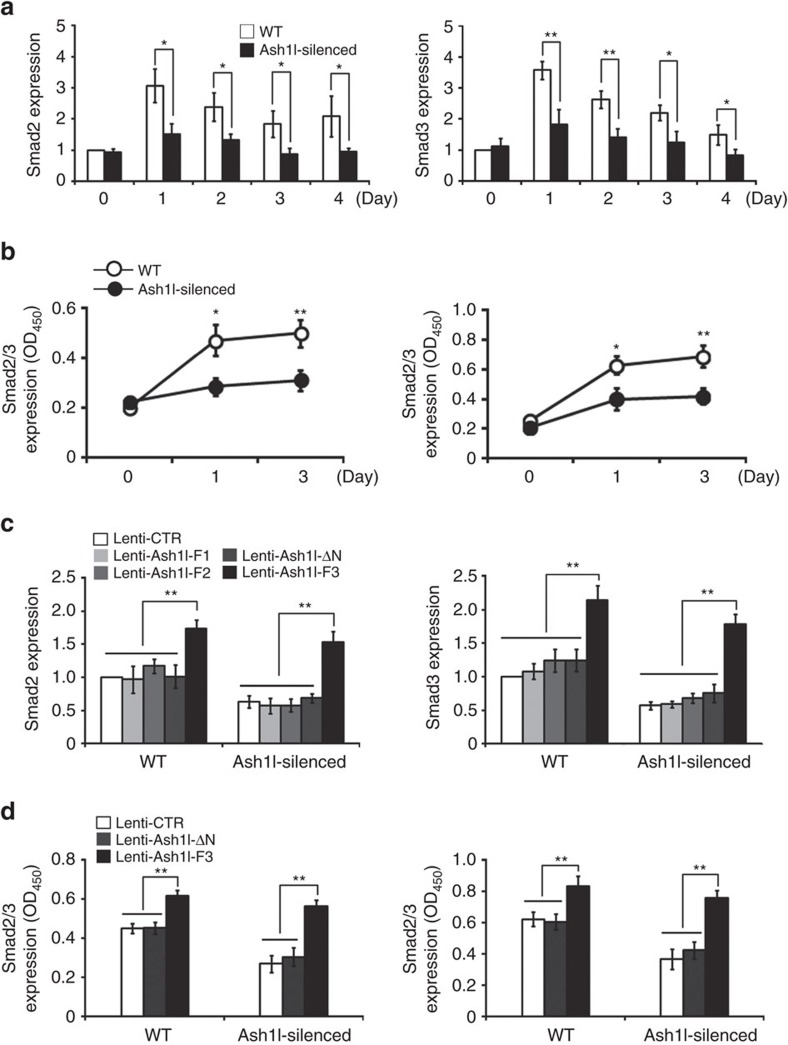
Ash1l enhances TGF-β-Smad2/3 signalling by promoting Smad2/3 expression. (**a**) mRNA expression of Smad2 (left) and Smad3 (right) in the WT and Ash1l-silenced CD4^+^ T cells stimulated under iTreg cell-skewing conditions (with TGF-β) for indicated times. Results are relative to those in unstimulated WT CD4^+^ T cells, set as 1. (**b**) ELISA assay of Smad2/3 in 5 μg whole-cell extract (left) and 5 μg nuclear extract (right) of WT and Ash1l-silenced CD4^+^ T cells under iTreg cell-skewing conditions (with TGF-β) for indicated times. OD_450_ represents absorbance at 450 nm. (**c**) mRNA expression of Smad2 and Smad3 in the WT and Ash1l-silenced CD4^+^ T cells transduced with a control lentivirus (Lenti-CTR) or Ash1l-fragment-expressing lentivirus (Lenti-Ash1l-F1, Lenti-Ash1l-F2, Lenti-Ash1l-F3 or Lenti-Ash1l-ΔN) and cultured under iTreg cell-skewing conditions (with TGF-β) for 3 days. Results are relative to those in WT CD4^+^ T cells transduced with Lenti-CTR, set as 1. (**d**) ELISA assay of Smad2/3 in 5 μg whole-cell extract (left) and 5 μg nuclear extract (right) of WT and Ash1l-silenced CD4^+^ T cells transduced and cultured as in **c**. OD_450_ represents absorbance at 450 nm. Error bars represent s.d. Student's *t* test. *P<0.05, **P<0.01. All data are from three independent experiments (mean±s.d. of technical triplicates).

The induction of iTreg cells is mainly mediated by the TGF-β-Smad signalling. To further investigate the mechanism of Ash1l-mediated promotion of iTreg cell polarization, we examined the expression and activation of Smad proteins in WT and Ash1l-silenced CD4^+^ T cells upon TGF-β stimulation. Compared to WT CD4^+^ T cells, lower mRNA levels of Smad2 and Smad3 were observed in Ash1l-silenced CD4^+^ T cells under iTreg cell-skewing condition (Fig. 3a). With ELISA assay, we also observed less Smad2/Smad3 expression in whole-cell extract and nuclear extract of Ash1l-silenced CD4^+^ T cells during iTreg cell polarization, further indicating impaired induction and activation of Smad2 and Smad3 (Fig. 3b). Thus, Ash1l promotes TGF-β-induced Foxp3 expression through enhancing TGF-β-Smad2/3 signalling.

Then, we observed that only overexpression of lentivirus Ash1l-F3 could increase the expression of Smad2 and Smad3, rescuing the impairment of Smad2/3 activation in Ash1l-silenced CD4^+^ T cells (Fig. 3c,d). The data indicated that Ash1l indeed facilitates TGF-β-Smad2/3 signalling via enhancing Smad2/3 expression dependent on its H3K4 methyltransferase activity of SET domain.

The above data promoted us to investigate the expression of ASH1L, FOXP3 and SMAD3 in T cells of the patients with inflammatory diseases. Indeed, ASH1L, FOXP3 and SMAD3 were significantly downregulated in CD4^+^ T cells from peripheral blood mononuclear cells (PBMCs) of patients with rheumatoid arthritis as compared to healthy controls (Supplementary Fig. 6; Supplementary Table 1), which indicates that ASH1L and ASH1L-mediated SMAD3/FOXP3 regulation might have potential significance in human autoimmune pathogenesis.

### Ash1l targets Smad2/3 promoters to induce Smad2/3 expression

**Figure 4:**
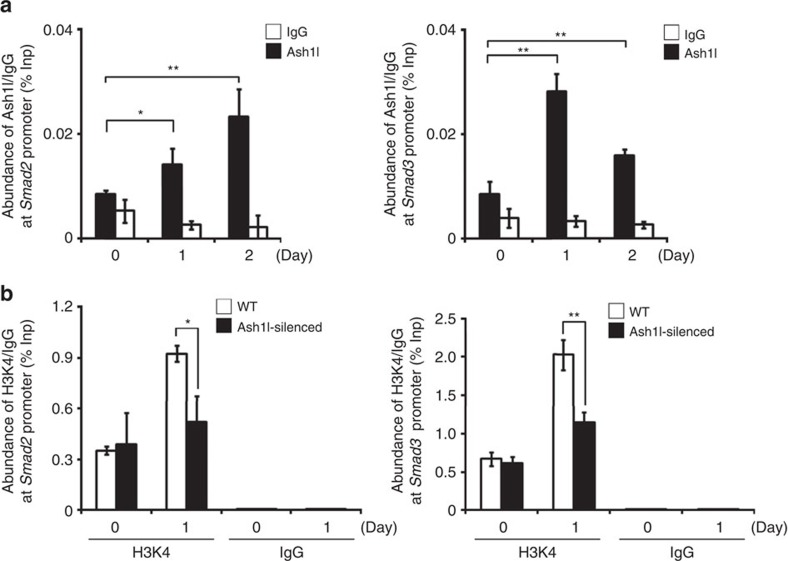
Ash1l accumulates at the *Smad2/3* promoter regions upon TGF-β stimulation. (**a**) CHIP analysis of the recruitment of Ash1l to the *Smad2* and *Smad3* promoter regions with anti-Ash1l antibody in CD4^+^ T cells stimulated under iTreg cell-skewing conditions (with TGF-β) for indicated times. Normalized data are shown as percentage of input control (% Inp). IgG serves as a CHIP control. (**b**) H3K4me3 modifications of the *Smad2* and *Smad3* promoter regions. CHIP analysis of the trimethylation of histone H3 lysine 4 (H3K4me3) at the *Smad2* and *Smad3* promoter regions in WT and Ash1l-silenced CD4^+^ T cells stimulated under iTreg cell-skewing conditions (with TGF-β) for 1 day. Normalized data are shown as percentage of input control (% Inp). IgG serves as a CHIP control. Error bars represent s.d. Student's *t* test. *P<0.05, **P<0.01. All data are from three independent experiments (mean±s.d. of technical triplicates).

Since Ash1l is a histone methyltransferase that functions to promote gene transcription^13^, we investigated whether Ash1l could directly upregulate Smad2/3 expression at transcriptional level. Chromatin immunoprecipitation (CHIP) experiments with antibody to Ash1l revealed that Ash1l was enriched at the *Smad2* and *Smad3* promoter regions in iTreg cells (Fig. 4a). Next, we assessed the H3K4 trimethylation (H3K4me3) modification at *Smad2* and *Smad3* promoter regions in Ash1l-silenced and WT CD4^+^ T cells under iTreg cell-skewing condition. CHIP experiments with antibody to H3K4me3 revealed that TGF-β-induced upregulation of H3K4me3 modification at the promoter regions of *Smad2* and *Smad3* was attenuated in Ash1l-silenced CD4^+^ T cells (Fig. 4b). And we observed that the enrichment of Ash1l at *Smad3* promoters was more potently upregulated by TGF-β stimulation than that at *Smad2* promoters (Fig. 4a). Similar to Ash1l-silenced mice, Smad3-KO mice showed normal natural T cell development but impaired iTreg cell polarization (Supplementary Fig. 7a–f).

In contrast, the level of Smad4 was comparable between Ash1l-silenced and WT CD4^+^ T cells during iTreg cell polarization (Supplementary Fig. 8a). Consistently, we did not observe enrichment of Ash1l at the *Smad4* and Foxp3 promoter regions in iTreg cells, nor did we see altered H3K4me3 modification at the *Smad4* and *Foxp3* promoter regions in Ash1l-silenced CD4^+^ T cells under iTreg cell-skewing condition (Supplementary Fig. 8b–e).

These data indicate that upon TGF-β stimulation, Ash1l selectively targets *Smad2* and *Smad3* promoter regions, and mediates H3K4me3 modification to enhance the expression of Smad2 and Smad3, thus strengthens TGF-β signalling.

### Smad3 suppresses lnc-smad3 to promote iTreg polarization

The data presented above confirmed that Ash1l directly enhanced Smad2/3 expression, next we tried to give some cues for explaining the Ash1l-mediated selective regulation on Smad proteins. Considering the more TGF-β-induced enrichment of Ash1l at *Smad3* promoter than that at *Smad2* promoter, so we focused on explaining the selective promotion on Smad3 expression by Ash1l.

**Figure 5:**
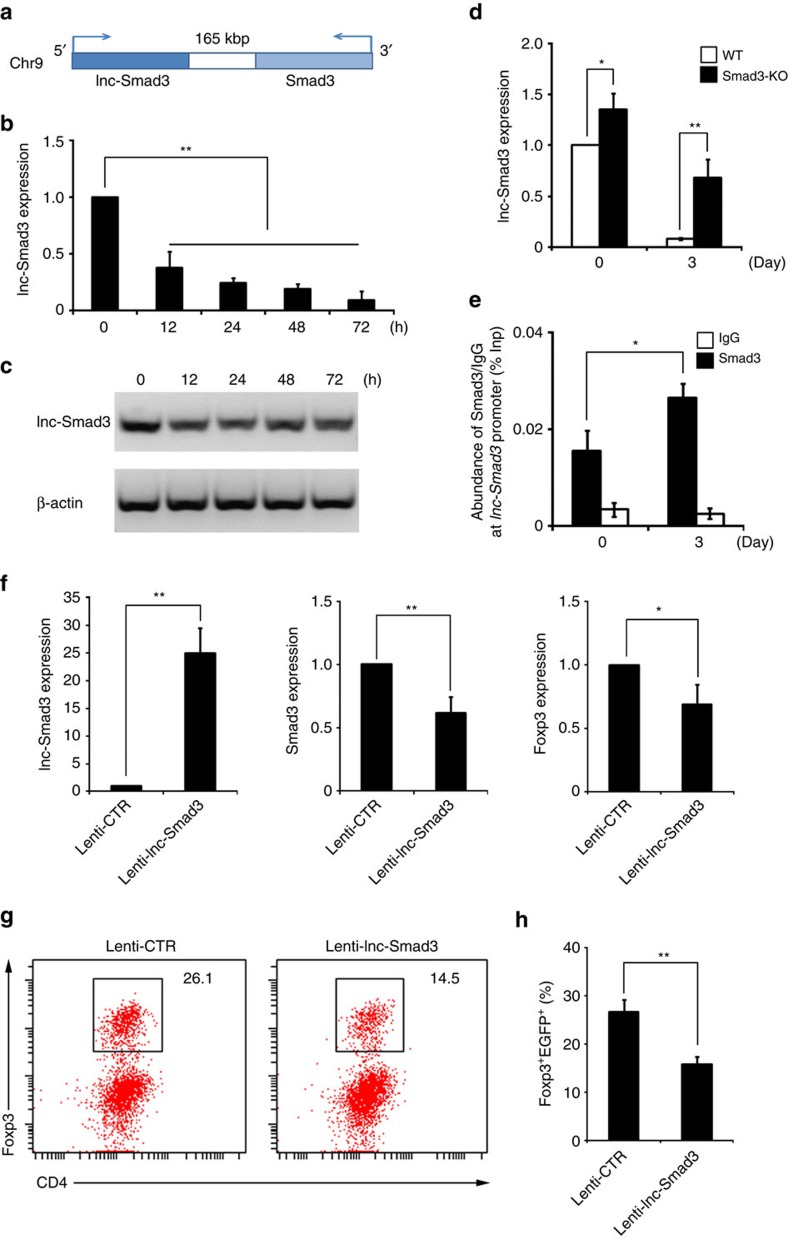
Lnc-Smad3 inhibits iTreg cell polarization via suppressing Smad3 transcription. (**a**) A schematic outlining the genomic loci of *lnc-Smad3* and *Smad3*. Semi-quantitative PCR (**b**) and gel electrophoresis (**c**) detection of lnc-Smad3 in CD4^+^ T cells stimulated under iTreg cell-skewing conditions (with TGF-β) for indicated times. Results are relative to the baseline lnc-Smad3 expression in unstimulated CD4^+^ T cells, set as 1. (**d**) Relative expression of lnc-Smad3 in WT and Smad3-KO CD4^+^ T cells stimulated under iTreg cell-skewing conditions (with TGF-β) for 3 days. Results are relative to the lnc-Smad3 expression in unstimulated WT CD4^+^ T cells, set as 1. (**e**) CHIP analysis of the accumulation of Smad3 at the *lnc-Smad3* promoter regions with anti-Smad3 antibody in CD4^+^ T cells stimulated under iTreg cell-skewing conditions (with TGF-β) for 2 days. Normalized data are shown as percentage of input control (% Inp). IgG serves as a CHIP control. (**f**) Relative expression of lnc-Smad3, Smad3 and Foxp3 in CD4^+^ T cells transduced with a control lentivirus (Lenti-CTR) or lnc-Smad3-expressing lentivirus (Lenti-lnc-Smad3) and cultured under iTreg cell-skewing conditions (with TGF-β) for 3 days. Results are relative to those in CD4^+^ T cells transduced with Lenti-CTR, set as 1. (**g**,**h**) The percentages of Foxp3^+^ Tregs in CD4^+^ T cells transduced and cultured as in **f** were analysed by flow cytometry (**g**) and quantified (**h**). Numbers in quadrants indicate per cent cells in each. Error bars represent s.d. Student's *t* test. NS, not significant. *P<0.05, **P<0.01. Data are from three independent experiments (**b**,**d**-**f**,**h**; mean±s.d. of technical triplicates) or are representative of three independent experiments (**c**,**g**).

lncRNAs have been increasingly shown to regulate diverse biological processes including T cell development through affecting the expression of neighbouring genes^19^. A recent study catalogued the lncRNA expression profiles in various T cell types including iTreg cells^20^. So, we sought to determine whether there were any unrecognized lncRNAs proximal to the *Smad3* gene locus that might be involved in Ash1l-mediated selective regulation of Smad3 expression and iTreg cell polarization. By analysing the gene loci nearby *Smad3* locus from UCSC genome browser, we found an lncRNA (Gene symbol GM16759) located 165 kb upstream of *Smad3* (Fig. 5a). We named this new lncRNA lnc-Smad3 because of its location and function verified as follows. We identified its sequence with 1,260 nucleotides (nt). Like most identified lncRNAs, lnc-Smad3 had 5′ cap structure and polyA tail, but had no protein coding capacity (Supplementary Fig. 9a,b). Lnc-Smad3 was expressed highly in CD4^+^ T cells and B cells, but extremely lowly in other immune cells such as CD8^+^ T cells and iTreg cells (Supplementary Fig. 9c). In addition, lnc-Smad3 was mainly located in the nucleus in CD4^+^ T cells, with a small portion existed in the cytoplasm (Supplementary Fig. 9d,e).

In contrast to the upregulation of Smad3 by TGF-β (Fig. 3a,b), the expression of lnc-Smad3 was gradually reduced after TGF-β stimulation in CD4^+^ T cells and B cells (Fig. 5b,c; Supplementary Fig. 9f). The inverse correlation between lnc-Smad3 and Smad3 expression during iTreg cell polarization and close location of these two genes prompted us to determine whether lnc-Smad3 and Smad3 regulate each other's expression. Since previous studies demonstrated Smad3 suppressed expression of some TGF-β-responsive genes via binding to their promoter regions^21^, we suspected Smad3 might directly target *lnc-Smad3* promoter region to inhibit its expression as a transcriptional suppressor. Indeed, Smad3-deficient CD4^+^ T cells had significantly increased lnc-Smad3 expression than WT CD4^+^ T cells, both before and more significantly after TGF-β administration, indicating that Smad3 negatively regulates lnc-Smad3 expression (Fig. 5d). CHIP assays with antibody to Smad3 revealed that TGF-β induced Smad3 accumulation at the *lnc-Smad3* promoter region (Fig. 5e). These results indicated that Smad3 acts as a transcriptional suppressor of lnc-Smad3 in TGF-β-mediated iTreg cells by directly targeting the *lnc-Smad3* promoter region.

Next, we investigated the potential function of lnc-Smad3 in TGF-β-induced Smad3 and Foxp3 expression and iTreg cell differentiation. Upon TGF-β stimulation, overexpression of lnc-Smad3 led to a reduction of Smad3 and Foxp3 expression and impairment in the iTreg cell polarization in CD4^+^ T cells (Fig. 5f–h). However, knockdown of lnc-Smad3 in activated CD4^+^ T cells neither affected the expression of Smad3 and Foxp3 nor polarized iTreg cell polarization without TGF-β stimulation (Supplementary Fig. 10a–c), thus the reduction of lnc-Smad3 is required for iTreg cell polarization in a manner dependent on TGF-β. Altogether, TGF-β-induced Smad3 inhibits lnc-Smad3 expression via binding to its promoter region as a suppressor, and downregulation of lnc-Smad3 mediated by TGF-β consequently skews CD4^+^ T cells towards iTreg cells via increasing Smad3 and Foxp3 expression.

### Lnc-Smad3 prevents Ash1l binding to the *Smad3* promoter

**Figure 6:**
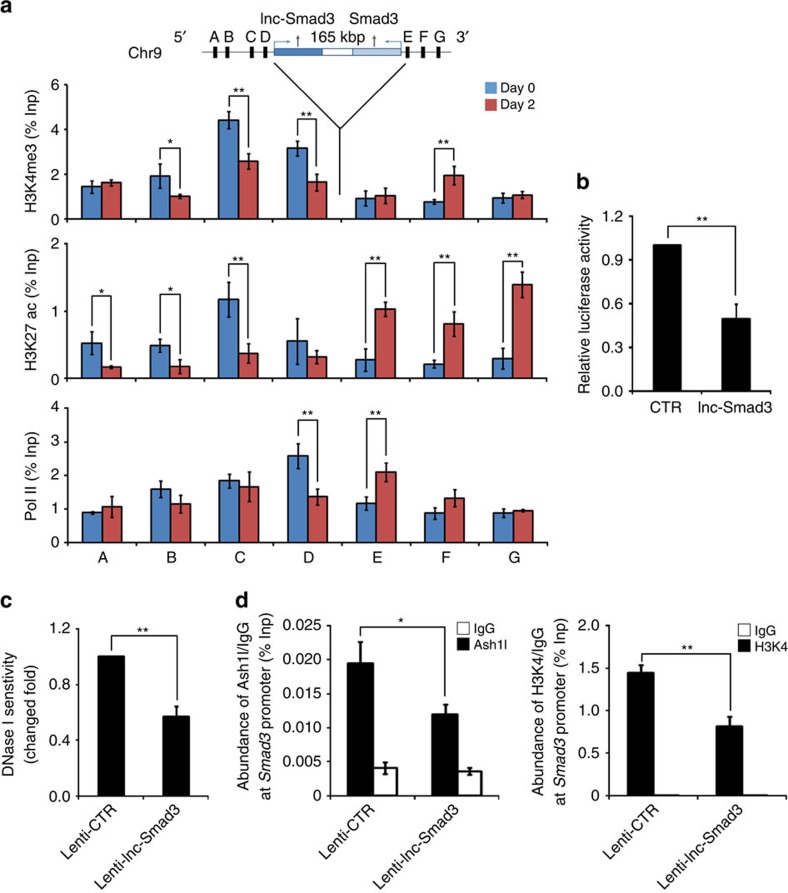
Lnc-Smad3 reduces the accessibility of the *Smad3* promoter to Ash1l. (**a**) CHIP analysis of the H3K4me3 and H3K27ac modifications, and Pol II occupancy around *lnc-Smad3* and *Smad3* loci in CD4^+^ T cells left unstimulated (day 0) or cultured under iTreg cell-skewing conditions (with TGF-β) for 2 days (day 2). Four regions (capital letters A–D) across *lnc-Smad3* gene locus and three regions (capital letters E–G) across *Smad3* gene locus were analysed by CHIP assay. Normalized data are shown as percentage of input control. (**b**) HepG2 cells were transfected with a *Smad3* promoter reporter construct (1 kb upstream of the transcription start site) and lnc-Smad3 expression vector (lnc-Smad3) or empty control vector (CTR), and stimulated with TGF-β. Luciferase activity was measured 48 h later. Data were normalized to renilla luciferase and presented with respect to CTR, set as 1. (**c**) Chromatin accessibility of the *Smad3* promoter region by quantitative PCR with DNase I pretreated nucleus of CD4^+^ T cells transduced with a control lentivirus (Lenti-CTR) or lnc-Smad3-expressing lentivirus (Lenti-lnc-Smad3) and cultured under iTreg cell-skewing conditions (with TGF-β) for 2 days. Changed fold are concluded using 2^ΔCt^ with respect to CD4^+^ T cells transduced with Lenti-CTR, set as 1. (**d**) CHIP analysis of the accumulation of Ash1l and H3K4me3 modification at *Smad3* promoter regions in CD4^+^ T cells transduced and cultured as in **c**. Normalized data are shown as percentage of input control (% Inp). IgG serves as a CHIP control. Error bars represent s.d. Student's *t* test. *P<0.05, **P<0.01. All data are from three independent experiments (mean±s.d. of technical triplicates).

Given the inverse expression of lnc-Smad3 and Smad3 during iTreg cell polarization, we next examined the epigenetic profile in lnc-Smad3 and Smad3 loci. With CHIP experiments, we found that with TGF-β stimulation, the H3K4me3 and H3K27 acetylation (H3K27ac), and the occupancy of Pol II were downregulated at *lnc-Smad3* promoter region whereas these parameters were upregulated at the *Smad3* promoter region (Fig. 6a). This was consistent with the downregulation of lnc-Smad3 and upregulation of Smad3 expression during iTreg cell polarization.

Above data suggested that Ash1l and lnc-Smad3 mediated opposing regulation of Smad3 expression, so we sought to determine the underlying mechanisms how Ash1l and lnc-Smad3 established an epigenetic balance between inhibition and activation of *Smad3* transcription. Since lncRNAs usually regulate neighbouring genes by recruiting specific chromatin remodelers^19^, we performed RNA-immunoprecipitation (RIP) assay to assess the interaction between lnc-Smad3 and Smad3 or Ash1l. However, we observed no obvious interactions either between lnc-Smad3 and Smad3 or between lnc-Smad3 and Ash1l, indicating that lnc-Smad3 regulates Smad3 expression independently on the direct interaction with Smad3 or Ash1l (Supplementary Fig. 11a). To determine the direct effect of lnc-Smad3 in regulating *Smad3* promoter, we transfected HepG2 cells with lnc-Smad3 expression vector or empty control vector, as well as *Smad3* promoter luciferase-reporter construct under TGF-β stimulation. The luciferase activity was lower in the presence of exogenous lnc-Smad3 than that with empty control (Fig. 6b), indicating that lnc-Smad3 suppresses the activity of *Smad3* promoter.

Notably, a DNase I sensitivity assay revealed that the chromatin accessibility of the *Smad3* promoter was significantly attenuated in TGF-β-stimulated CD4^+^ T cells upon overexpression of lnc-Smad3 (Fig. 6c), indicating that lnc-Smad3 was involved in maintenance of the compact chromatin structure of the *Smad3* promoter. We went further to determine whether lnc-Smad3 regulates the binding of the *Smad3* promoter by Ash1l. With CHIP experiments using antibodies to Ash1l and H3K4me3, we found that overexpression of lnc-Smad3 in CD4^+^ T cells under TGF-β stimulation reduced the binding of Ash1l at the *Smad3* promoter region, and hence attenuated its H3K4me3 modification (Fig. 6d). In contrast, the chromatin accessibility, Ash1l binding and H3K4me3 modification of the *Foxp3* promoter were not altered by overexpression of lnc-Smad3 (Supplementary Fig. 11b,c), indicating that lnc-Smad3 selectively regulated the chromatin state of *Smad3* but not *Foxp3* promoter region.

**Figure 7:**
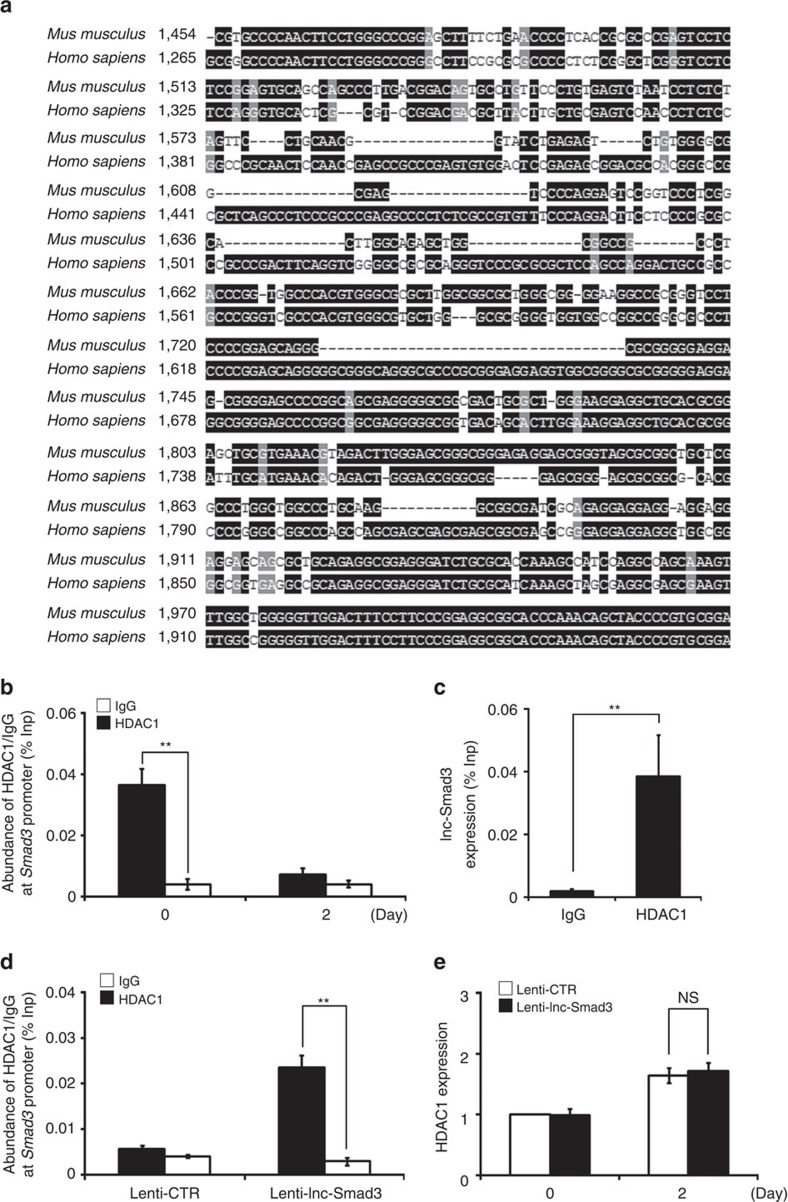
Lnc-Smad3 interacts with HDAC1 at the *Smad3* promoter. (**a**) A schematic outlining the conserved sequences of the *Smad3* promoter regions in *Mus musculus* and *Homo sapiens*. (**b**) CHIP analysis of the accumulation of HDAC1 at *Smad3* promoter region in naive CD4^+^ T cells and iTreg cells. Normalized data are shown as percentage of input control (% Inp). IgG serves as a CHIP control. (**c**) Quantitative PCR detection of the lnc-Smad3 retrieved by HDAC1 specific antibody compared with immunoglobulin G (IgG) in the RIP assay within CD4^+^ T cells. Normalized data are shown as percentage of input control (% Inp). IgG serves as a RIP control. (**d**) CHIP analysis of the accumulation of HDAC1 at *Smad3* promoter region in CD4^+^ T cells transduced with a control lentivirus (Lenti-CTR) or lnc-Smad3-expressing lentivirus (Lenti-lnc-Smad3) and cultured under iTreg cell-skewing conditions (with TGF-β) for 2 days. Normalized data are shown as percentage of input control (% Inp). IgG serves as a CHIP control. (**e**) Relative expression of HDAC1 in CD4^+^ T cells transduced and cultured as in **d**. Results are relative to the baseline expression of HDAC1 in unstimulated CD4^+^ T cells transduced with Lenti-CTR, set as 1. Error bars represent s.d. Student's *t* test. **P<0.01. NS, not significant. All data are from three independent experiments (mean±s.d. of technical triplicates).

Next, we tried to determine how lnc-Smad3 regulated the chromatin state of *Smad3* promoter region. Previous research demonstrated that HDAC1, a histone deacetylase, could bind to *Smad3* promoter region in lung cancer cells to suppress Smad3 expression in human lung cancer cells^22^. So, we investigated whether HDAC1 may have similar effect in mouse CD4^+^ T cells to close the chromatin structure of *Smad3* promoter. By homologous comparison, we found that the promoter regions of Smad3 were highly conserved between human and mouse (Fig. 7a). With CHIP experiments using antibody to HDAC1, we found HDAC1 bound to *Smad3* promoter in mouse naive CD4^+^ T cells, and TGF-β stimulation, which led to reduction of lnc-Smad3 expression (Fig. 5b), also prevented the accumulation of HDAC1 to *Smad3* promoter (Fig. 7b). With RIP assay, we observed the interaction between HDAC1 and lnc-Smad3 in naive CD4^+^ T cells (Fig. 7c). Furthermore, overexpression of lnc-Smad3 in CD4^+^ T cells under TGF-β stimulation increased the binding of HDACl at the *Smad3* promoter region (Fig. 7d) without affecting the HDAC1 expression (Fig. 7e). Thus, lnc-Smad3 selectively promoted the closed chromatin state of the *Smad3* promoter by recruiting HDAC1.

Collectively, our data suggest that lnc-Smad3 recruits HDAC1 to *Smad3* promoter region and suppresses Smad3 transcription, while TGF-β-induced Smad3 reduces lnc-Smad3 expression via accumulating at *lnc-Smad3* promoter regions. TGF-β-mediated reduction of lnc-Smad3 relieves its suppression on *Smad3* promoter region, and hence allows the accumulation of Ash1l at *Smad3* promoter regions to induce its H3K4me3 modification. Increased induction of Smad3 by Ash1l subsequently facilitates Foxp3 expression, contributing to the polarization of Treg cells and prevention of autoimmunity (Supplementary Fig. 12).

## Discussion

H3K4 histone methylation is one of the most characterized epigenetic modifications, and some associated enzymes have emerged to be critical regulators of cell development especially in hematopoiesis^23^,^24^,^25^. H3K4 methyltransferase Smyd3 has been shown to target *Foxp3* promoter region to increase its expression, promote iTreg cell development and protect mice from exacerbated inflammation during respiratory syncytial virus infection by a TGF-β-Smad3-dependent mechanism^26^. However, the functions of other H3K4 histone modifying enzymes in Treg cell differentiation remain poorly understood. In previous work, we have identified Ash1l, one of the H3K4 methyltransferases, as a negative regulator of innate inflammatory immune response^13^. Here, we demonstrate that Ash1l enhances TGF-β-induced Smad3 and Foxp3 expression and subsequently promotes iTreg cell polarization. Our data identify a new epigenetic modifier in Treg cell development and expand the function of Ash1l in immunity. Furthermore, ASH1L, SMAD3 and FOXP3 are all downregulated in CD4^+^ T cells from PBMCs of patients with rheumatoid arthritis, indicating the potential significance of ASH1L/SMAD3/FOXP3 pathway in human autoimmune pathogenesis.

Notably, whether the human Foxp3^+^ T cells induced by TGF-β stimulation *in vitro* could physiologically represent *in vivo* functional Treg cells are still controversial. Although TGF-β could induce Foxp3 expression both in human and mouse CD4^+^ T cells, the induced human Foxp3^+^ cells are functionally heterogeneous, with non-regulatory subpopulations showing little *in vitro* suppressive activity and even secrete proinflammatory cytokines such as IFN-γ^27^. Highly specific Treg cell surface markers in addition to Foxp3 are required to identify and purify the regulatory subpopulations in humans. The feasibility of using iTreg cells as a therapeutic intervention in human diseases is also limited by the stability of infused Treg cells^27^. Considering the possible plasticity of Treg cells towards other pathogenic T cell subsets such as Th17 cells, it is necessary to confirm the purity and stability of Treg cells during Treg cell-based therapy in humans. In addition, the detailed molecular mechanisms of Treg cell-mediated suppression in humans still remain to be determined. Altogether, it requires further investigation to clarify the function of Ash1l in human Treg cell development and human inflammatory immune diseases, especially via the improved methods for induction of immunosuppressive, purified and stable human iTreg cells.

Interestingly, unlike Smyd3, we find that Ash1l selectively targets *Smad3* promoter region but not that of *Foxp3*. Ash1l promotes Foxp3 expression indirectly via enhancing the expression of its upstream molecule Smad3. The TGF-β-induced Smad3 acts as an important transcriptional factor to promote Foxp3 expression^6^,^7^. Vitamin A metabolite retinoic acid can enhance the expression and phosphorylation of Smad3, subsequently leading to an increase in Foxp3 expression^28^. The β-galactoside-binding protein galectin-9 acts synergistically with TGF-β signalling to enforce iTreg cell differentiation and maintenance via forming a positive feedback loop with Smad3 (ref. 29). TGF-β stimulation in T cells can induce Smad3 activation by phosphorylation, however, the epigenetic mechanisms governing TGF-β-dependent Smad3 expression is still unclear. By identifying the chromatin modifier Ash1l as a positive regulator of Smad3, we expand the epigenetic regulation network of TGF-β-Smad signalling and iTreg cell polarization.

lncRNAs have emerged as key regulators in multiple biological processes such as immune response, hematopoiesis, oncogenesis and so on^30^,^31^,^32^,^33^. We previously showed lnc-DC is selectively expressed in human dendritic cells and is required for the maturation and activation of dendritic cells^34^. Some T helper cell-specific lncRNAs have been identified and shown to regulate T cell differentiation. For example, linc-MAF-4 suppresses the Th2 polarization by associating with the chromatin modifiers LSD1 and Ezh2 (ref. 19). Another lncRNA, lincR-Ccr2-5′AS is identified as a Th2-specific lncRNA, which enhances the expression of numerous Th2-associated genes and promotes Th2 migration to lung tissue^20^. Rmrp, an lncRNA associated with human cartilage-hair hypoplasia, was shown to regulate the transcriptional expression of RORγt target genes, hence controlling the differentiation of Th17 cells and development of inflammatory diseases^35^. However, until now, lncRNAs that specifically regulate Treg cell polarization are still unknown. Here, we identified an lncRNA, named as lnc-Smad3, whose expression is inversely correlated with that of Smad3, is involved in suppression of iTreg cell induction via inhibiting TGF-β-mediated Smad3 expression.

Various lncRNAs act as modular scaffold or recruiter of histone modification complexes^36^,^37^. In our study, we found Lnc-Smad3 is involved in kept of the compact chromatin structure of the *Smad3* promoter by interacting with HDAC1. The downregulation of lnc-Smad3 induced by TGF-β signalling hence allows Ash1l to be recruited and then activate the transcription of Smad3. Thus, during TGF-β-mediated iTreg cell polarization, lnc-Smad3 does not act as a scaffold or recruiter of Ash1l but functions as a guard of *Smad3* promoter region via preventing the accumulation of Ash1l. Lnc-Smad3 regulates the chromatin state of *Smad3* promoter mainly in the nucleus. However, a small portion of lnc-Smad3 is also detected in the cytoplasm. The biological function of cytoplasmic lnc-Smad3 awaits further investigations. It's also intriguing to find out whether additional lncRNAs or chromatin modifiers might directly interact with Ash1l and recruit it specifically to *Smad3* promoter region.

Taken together, our research identifies Ash1l and lnc-Smad3 as two opposing critical epigenetic modifiers that regulate TGF-β-mediated Treg cell polarization. We provide new insights into the epigenetic modifications of Treg cell polarization and immune homoeostasis and suggest possible clues for intervention of systemic inflammation and autoimmune diseases.

## Methods

### Mice

Ash1l-silenced mice on FVB background were from Dr Tian Xu (Institute of Developmental Biology and Molecular Medicine, Fudan University), and were backcrossed (12 polarizations) onto the C57BL/6J background^13^. Rag1^−/−^ mice on the C57BL/6 background were from Shanghai Laboratory Animal Center, Chinese Academy of Science. Smad3-KO mice were kindly provided by Dr Xiao Yang (Laboratory Animal Center, Academy of Military Medical Science); CD45.1^+^ mice were from The Jackson Laboratory (B6.SJL-*Ptprc*^*a*^ *Pepc*^*b*^/BoyJ; 002014). All mice were maintained under specific pathogen-free conditions and used at 6–8 weeks of age. All animal experiments were approved by the Scientific Investigation Board of Second Military Medical University, Shanghai.

### Human subjects

A total of 10 rheumatoid arthritis (RA) and 9 healthy control (HC, with no history of autoimmune disease) patients were recruited from Peking Union Medical College Hospital and Peking University People's Hospital and their peripheral blood samples were prepared from patients after informed consent was provided^38^. RA was diagnosed according to the criteria of the American College of Rheumatology. The clinical characteristics of the individuals are shown in Supplementary Table 1. The study was approved by the Research Ethics Boards of Peking Union Medical College Hospital and Peking University People's Hospital.

### Reagents

Anti-mouse CD11c, CD8 and B220-coated magnetic beads were from Miltenyi Biotech. Recombinant mouse GM-CSF and IL-4 were from Peprotech EC. APC-conjugated anti-mouse Foxp3 (FJK-16s), GITR (DTA-1), IFN-γ (XMG1.2), CTLA-4 (UC10-4B9), FITC-conjugated anti-mouse CD8a (53-6.7), IL-4 (BVD6-24G2), IL-10 (JES5-16E3), and anti-IFN-γ (R4-6A2), anti-IL-4 (11B11) mAbs were from eBioscience. FITC-conjugated anti-mouse Foxp3 (MF23), CD25 (7D4), PE-Cy7-conjugated anti-mouse CD4 (RM4-5), APC-conjugated anti-mouse CD62L (MEL-14), and anti-CD3 (145-2C11), anti-CD28 (37-51) mAbs, Golgi Plug were from BD PharMingen. FITC-conjugated anti-mouse CD44 (IM7), APC-conjugated IL-17A (TC11-18H10), recombinant mouse IL-2, IL-6 and IL-12 were from BioLegend. Recombinant human TGF-β was from R&D Systems. Antibody specific to Ash1l (sc-98301X) was from Santa Cruz. Antibody specific to HDAC1 (ab7028) was from Abcam. The antibodies for cell staining were used at recommended dilution rates (1:1,000) according to the manufacturers' instructions. TRIzol reagents were from Invitrogen. PrimeScript RT-PCR Kit (2641A) and SYBR Premix ExTaq Kit (RR420) were from Takara Bio Inc. RPMI1640 medium and FBS (fetal bovine serum) were from PAA laboratories.

### Human PBMC isolation and CD4^+^ T cells sorting

Human PBMCs were collected and purified by Ficoll-Paque PLUS (GE healthcare) density gradient centrifugation. In brief, collected whole-blood samples were diluted and mixed with Hanks' balanced salt solution (HBSS). Then blood mixture was slowly added to layer over Ficoll. PBMCs were collected at the interface of the upper layer and the Ficoll after centrifugation (400*g*, 30 min). CD4^+^ T cells were sorted from fresh PBMCs with PerCP-conjugated anti-human CD4 antibody (Biolegend, 317432) by FACS Aria II flow cytometer (BD Biosciences). The FACS sorting strategy was introduced in Supplementary Fig. 13.

### T cell isolation and polarization

Naive CD4^+^ T cells were purified from splenocytes, mLN (mesenteric lymph nodes) or thymus with mouse naive CD4^+^ T cell isolation kit (Stemcell, 19765) and cultured under neutral (Th0) conditions with anti-CD3 (1 μg ml^−1^), soluble anti-CD28 mAbs (1 μg ml^−1^) and 10% (vol/vol) FBS in RPMI1640, unless otherwise indicated. For T cell polarization, CD4^+^ T cells were cultured in the neutral medium described above and stimulated with polarizing cytokines as follows: for iTreg, anti-IFN-γ (10 μg ml^−1^), anti-IL-4 (10 μg ml^−1^), IL-2 (5 ng ml^−1^) and recombinant human TGF-β (10 ng ml^−1^); for Th1, anti-IL-4 (10 μg ml^−1^), IL-2 (5 ng ml^−1^) and IL-12 (10 ng ml^−1^); and for Th17, anti-IFN-γ (10 μg ml^−1^), anti-IL-4 (10 μg ml^−1^), human IL-6 (10 ng ml^−1^) and recombinant human TGF-β (1 ng ml^−1^). Differentiated iTreg cells were further enriched with mouse CD25 positive selection kit (Stemcell, 18761).

### *In vitro* suppression assay

A total of 5 × 10^4^ carboxyfluorescein diacetate succinimidyl ester (CFSE)-labeled naive CD4^+^ effector T cells from CD45.1 mice were cultured with 1 × 10^4^ CD11c^+^ DC used as antigen-presenting cells in 96-well round-bottom plates. Overall, 2.5 × 10^4^ differentiated and purified WT or Ash1l-silenced iTreg cells were added as suppressive cells. Cells were stimulated with anti-CD3 (1 μg ml^−1^) for 3 days. T cell proliferation was determined by CFSE dilution with flow cytometry.

### Induction of colitis

Mice were presensitizated with 150 μl of 1% (w/v) TNBS solution diluted in acetone/olive oil (Sigma-Aldrich, MO, USA) on the back. On day 7, mice were further given 100 μl of 2.5% (w/v) TNBS solution (diluted in 50% ethanol) or ethanol only intrarectally through a catheter inserted into the colon 4 cm proximal to the anus. Beginning from the second sensitization, inflammatory bowel disease development was monitored and recorded by measuring the body weight daily. Mice were killed 3 days after the second sensitization and subjected to examination of colon morphology and histology, as well as Treg cells proportion. T cell adoptive transfer colitis was performed as follows. Rag1^−/−^ mice were injected intravenously with 4 × 10^5^ CD4^+^CD25^−^ cells from CD45.1 WT mice with or without 1 × 10^5^ WT or Ash1l-silenced CD4^+^CD25^+^ differentiated iTreg cells. Weight changes were monitored weekly. Mice were killed at week 6 and subjected to examination of colon morphology and histology.

### *In vivo* iTreg induction

Rag1^−/−^ mice were injected intravenously with 4 × 10^5^ CD4^+^CD25^−^ cells from WT or Ash1l-silenced mice. Monitored the weight changes and scarified the mice at week 4. T cells were isolated and Foxp3 expression was analysed with flow cytometry.

### Flow cytometry

For cell surface staining, the single-cell suspensions were incubated with the antibody cocktails for 20 min at 4 °C. For intracellular staining, cells were fixed and permeabilized with 4% paraformaldehyde for 20 min at 4 °C followed by intracellular staining with specific anti-cytokine antibodies. Data were obtained on an LSR II and analysed with FACSDiva software (both from BD Biosciences). The FACS gating strategies were represented in Supplementary Fig. 13.

### Quantitative real time PCR assay

SYBR RT-PCR kit (Takara) and LightCycler (Roche) were used for quantitative RT-PCR analysis^39^. Data were normalized to β-actin expression. Sequences of the primers for quantitative real time RT-PCR are in Supplementary Table 2.

### Nucleus and cytoplasm fraction isolation

Cells were lysed in 0.1% NP40 ice-cold PBS with protease inhibitor cocktail (Calbiochem, La Jolla, CA, USA) and Ribonucleoside Vanadyl Complex (10 mM) (New England BioLabs), then after short centrifugation, the supernatant was collected as cytoplasmic fraction and the remainder with additional washing were considered as nuclear pellets.

### Smad2/3 ELISA assay

The activated Smad2/3 in nuclear extract or the whole-cell lysate was detected with Smad2/3 ELISA Kit according to the manufacture's protocol (Signosis, CA, USA, TE-0011). In brief, 5 μg nuclear extract or the whole-cell lysate was added in the 96-well clear plate pre-immobilized with the Smad2/3 consensus sequencing oligo, incubated and washed. Then the activated Smad2/3 binding to the oligo was detected with a specific antibody against Smad2/3 subunit and a HRP-conjugated secondary antibody. The optical density of each well was determined with a microplate reader at 450 nm.

### Plasmid constructions

Recombinant vectors encoding mouse Smad3 (NM_016769.4) were constructed by PCR-based amplification from cDNA of mouse CD4^+^ T cells and then subcloned into the pcDNA3.1 eukaryotic expression vector (Invitrogen). Lnc-Smad3, Ash1l-fragment 1, 2, 3, and Ash1l-fragment3 mutant expression vectors were constructed by PCR-based amplification from cDNA of mouse CD4^+^ T cells and then subcloned into pCDH cDNA Cloning and Expression Lentivectors (System Biosciences). All constructs were confirmed by DNA sequencing. These plasmids transfected into HEK293T cell line (from American Type Culture Collection) with JetPEI reagents (Polyplus), and the protein coding capacity was tested by western blotting assay. The *Smad3* promoter luciferase reporter was constructed via cloning the fragment of the *Smad3* promoter (1,000 bp upstream of transcription start site) into the pGL3 luciferase-reporter vector (Promega) and the DNA sequence of the insert was verified.

### Reporter assay

HepG2 cells (3 × 10^4^ cells per well, 96-well plate, from American Type Culture Collection) were transiently transfected with the lnc-Smad3 expression vector, or empty control vector as well as the *Smad3* promoter firefly luciferase-reporter construct and Renilla luciferase-reporter vector (Promega) with Fugene HD (Promega). Cells were stimulated with TGF-β 24 h after transfection. Twenty-four hours after stimulation luciferase expression was determined by measuring luminescence with the Dual-Luciferase Reporter Assay System (Promega). The firefly luciferase activity was normalized to renilla luciferase activity.

### Knockdown and overexpression

Naive CD4^+^ T cells were stimulated with plate-coated anti-CD3 (1 μg ml^−1^), soluble anti-CD28 mAbs (1 μg ml^−1^) and IL-2 (10 ng ml^−1^) for 24 h before knockdown or overexpression assay. For knockdown assay, the target sequence of lnc-Smad3 was 5′-CATTGGACCATTTGATTCTTCCTAA-3′ (bolded and underlined in the following lnc-Smad3 sequence); the control siRNA sequence was 5′-CATCCAGTTTATTAGCTTCCGTTAA-3′. siRNA was transfected into activated CD4^+^ naive T cells through the use of Lipofectamine 2000 according to the manufacture's protocol (Life Technologies). For overexpression experiments, virus solutions containing lentivirus and polybrene (8 μg ml^−1^) were added to the activated CD4^+^ T cells, followed by centrifugation at 2,500 r.p.m for 2 h at room temperature. Virus supernatants were replaced with fresh culture medium at 6 h after infection. Cells were cultured for an additional 18 h and then sorted as enhanced green fluorescent protein (EGFP)-positive cells. Sorted cells were cultured for 3 days under iTreg cell conditions. Transduction efficiency was determined by EGFP expression and knockdown efficiency was measured by RT-PCR.

>mouse lnc-Smad3 transcript (GenBank Accession number: KY652933) CGGCGCGTGCGCACAGGACGGGACGGGAGGGCGGAGCGACTGCGCAGATCAGGAAAATTGTCACCTCTGGCCTCAGGGAAACTGAGGCTCTGAGCAGTTAAGAGGCCAACGATCCAGGTTTATGCTATCAGTGTCTGAGATACAATTAAGTCACCTTTTTGGGTGACATTTCCCTTGACTAGTACCTCAAGATTATGATCCAGGTGGACCATCCCTCCATTGCCTCCAGACATGTCTATGACGCTACCTAGGGATTGTGAAGATTTACCACCTGGTGGAAAATTAAAAAAAAAGACTTATTCTGCAAATTGACCAAGCTTAAGAGATACAGCACTGAGAATTCACCCACTACAGAGCTGGTAACCAGGCCTTCAGAGTAAGCTGTGATGTATAGCCATTCTCCTCAGCAGCCTGTTTGACTGAGGGATATAGGAATGACTGCCCTTACCCATGTTTTTGTTCAGATTTATGGTTCTAAATCTGATGGAAAATCTAT**CATTGGACCATTTGATTCTTCCTAA**AGAAAGGCCAAACCACCTGTCAAACCCACGAGCAACACAGCAATGGCTAAGCTGAAGAAGCCAAGTTATATGTCTAGCTCCTCTGTGGGTGGCAGGATCCAGCTGTGGGGAGTCCAAGGTTCCTTGTGTTCTTCTGGCTTGACTATGATATTTCCAATTTGGAGGCACAAAGAATTGAGAACTTGGGGATCCACTGAGGTCTGAGGCATGGTGGTACCAGAAGAAATAAAGGGGCCATCAGCATGAAGCTGGTCTCCCATTGACAGCACCCTGACTTCTCCATCAGGTTCTATCAGCATGTCTACTGTGAGGTTGGTGACGCTGTCTGCAGGTGGGTATGCTTCAATCACGCCACCTGGTCAGGAAACCATGGAGTAGAGGAAAAGACACAGTCAAGGGAATGCTATGCTTATAGCAGGAGGAAATGCTCTGTGTTTTAGCAAGGGAACTTAGACTATCTCACACCTGTCATATCCTGGGAAACTTTGGGTGAGTAGTTCAACCTTTGGCCTCCTTTTCCTTATTTATAGAATGGAGGCATAATATCTACCTTGCCAAGCTTTTGTGAAGTTTGAGAAACTGAGTCTATGGTATTTAGCCTGGTACTTGGAACATGGGAGGGCTCAATGAACAGTAAGTAGTCTTTATTATGATCATAATTACAAATATTACAAATACTTTATAAAAGCCATACTTAGAACAACTCATTACGTTAATAAAGAATCCAAGGATTGTGGTC

### Chromatin immunoprecipitation

CHIP analyses were performed with the following antibodies: RNA Polymerase II (Pol II), acetyl-Histone H3 (Lys27), trimethyl-Histone H3 (Lys4), Ash1l and HDAC1. Fold enrichment was quantified using quantitative RT-PCR and calculated as a percentage of Input chromatin (% input). Sequences of the primers for amplification of the Smad2, Smad3, Smad4, Foxp3 and lnc-Smad3 promoter regions are in Supplementary Table 3.

### Chromatin accessibility analysis

For chromatin accessibility analysis, nucleus were pretreated with DNase I (0.1 U l^−1^, Promega) at 37 °C for 30 min and then stopped by EDTA (50 mM). Genome DNA was extracted and subjected to quantitative RT-PCR. Data were presented as changed fold concluded with 2^ΔCt^, with relative to CD4^+^ T cells transduced with Lenti-CTR, set as 1.

### Rapid amplification of cloned cDNA ends

Total RNA extracted from mouse B220^+^ B cells was subjected to RACR PCR with SMARTer RACE 5′/3′ Kit according to the manufacture's protocol (Clontech, CA, USA, 634558).

### RNA immunoprecipitation

RNA immunoprecipitation (RIP) was performed with antibodies specific to mouse Ash1l, Smad3 and HDAC1 by using Magna RIP RNA-Binding Protein Immunoprecipitation Kit according to the manufacture's protocol (Millipore, CA, USA, 17-700).

### RNA fluorescence in situ hybridization

Fluorescence-conjugated lnc-Smad3 probes were used for FISH assay. Naive CD4^+^T cells were fixed in 4% formaldehyde plus 10% acetic acid in PBS for 15 min at room temperature, and then were permeabilized in PBS plus 0.2%–0.5% Triton X-100 and 5 mM vanadyl ribonucleoside complex (10 mM) (New England BioLabs) for 5 min on ice, washed in PBS three times and rinsed once in 2 × SSC buffer^34^. Hybridization was carried out using DNA probe sets according to the protocol of Biosearch Technologies. Cells were observed with a Leica TCS SP2 confocal laser microscopy.

### Statistical analysis

The statistical significance between two groups was determined by Student's *t* test. *P* values of <0.05 were considered statistically significant (*P<0.05, **P<0.01).

### Data availability

All the data supporting the findings of this study are available within the article and its Supplementary Information files and from the corresponding author on reasonable request.

## Additional information

**How to cite this article:** Xia, M. *et al*. Ash1l and lnc-Smad3 coordinate *Smad3* locus accessibility to modulate iTreg polarization and T cell autoimmunity. *Nat. Commun.* **8**, 15818 doi: 10.1038/ncomms15818 (2017).

**Publisher's note:** Springer Nature remains neutral with regard to jurisdictional claims in published maps and institutional affiliations.

## Supplementary Material

Supplementary Information

Peer Review File
