# Supplementary material for: Ash1l and lnc-Smad3 coordinate Smad3 locus accessibility to modulate iTreg polarization and T cell autoimmunity
Source: Nat Commun. 2017 Jun 9;8:15818. doi: 10.1038/ncomms15818 (PMC5472765; doi:10.1038/ncomms15818)
Supplement: Supplementary Information [file ncomms15818-s1.pdf]

Type of file: pdf
Size of file: 0 KB
Title of file for HTML: Supplementary Information
Description: Supplementary Figures and Supplementary Tables

Type of file: pdf
Size of file: 0 KB
Title of file for HTML: Peer Review File
Description:

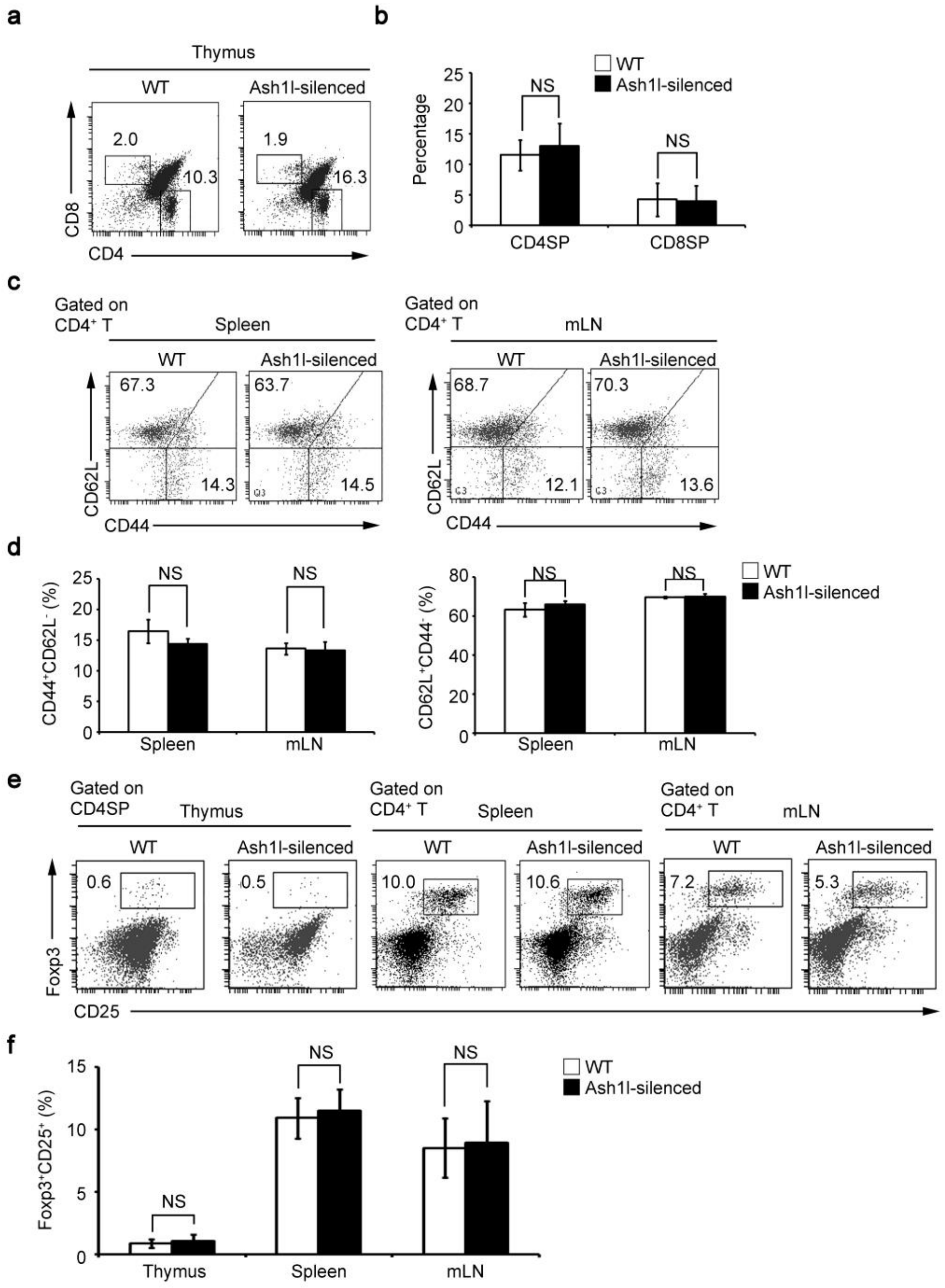

Supplementary Figure 1. Silencing of Ash1l does not affect T cell development.

(**a-d**) Flow cytometry profiles the thymus, spleen and mLN (mesenteric lymph nodes) from WT and Ash1l-silenced mice (n = 3 mice per group), showing the proportion of $CD4^+$ single-positive (CD4SP) and CD8SP thymocytes (**a, b**), and the expression of CD44 and CD62L in peripheral $CD4^+$ T cells (**c, d**). Numbers besides outlined areas or in quadrants indicate percent cells in each throughput.

(**e, f**) Flow cytometry profiles the thymus, spleen and mLN from WT and Ash1l-silenced mice (n = 3 mice per group), showing expression of Foxp3 in the CD4SP thymocytes and $CD4^+$ T cell fraction in the periphery. Numbers besides outlined areas indicate percent cells in each.

Error bars represent SD. Student's t test. NS, not significant. Data are representative of three independent experiments (**a**, **c, e**) or are from three independent experiments (**b, d, f**; mean ± SD of three mice).

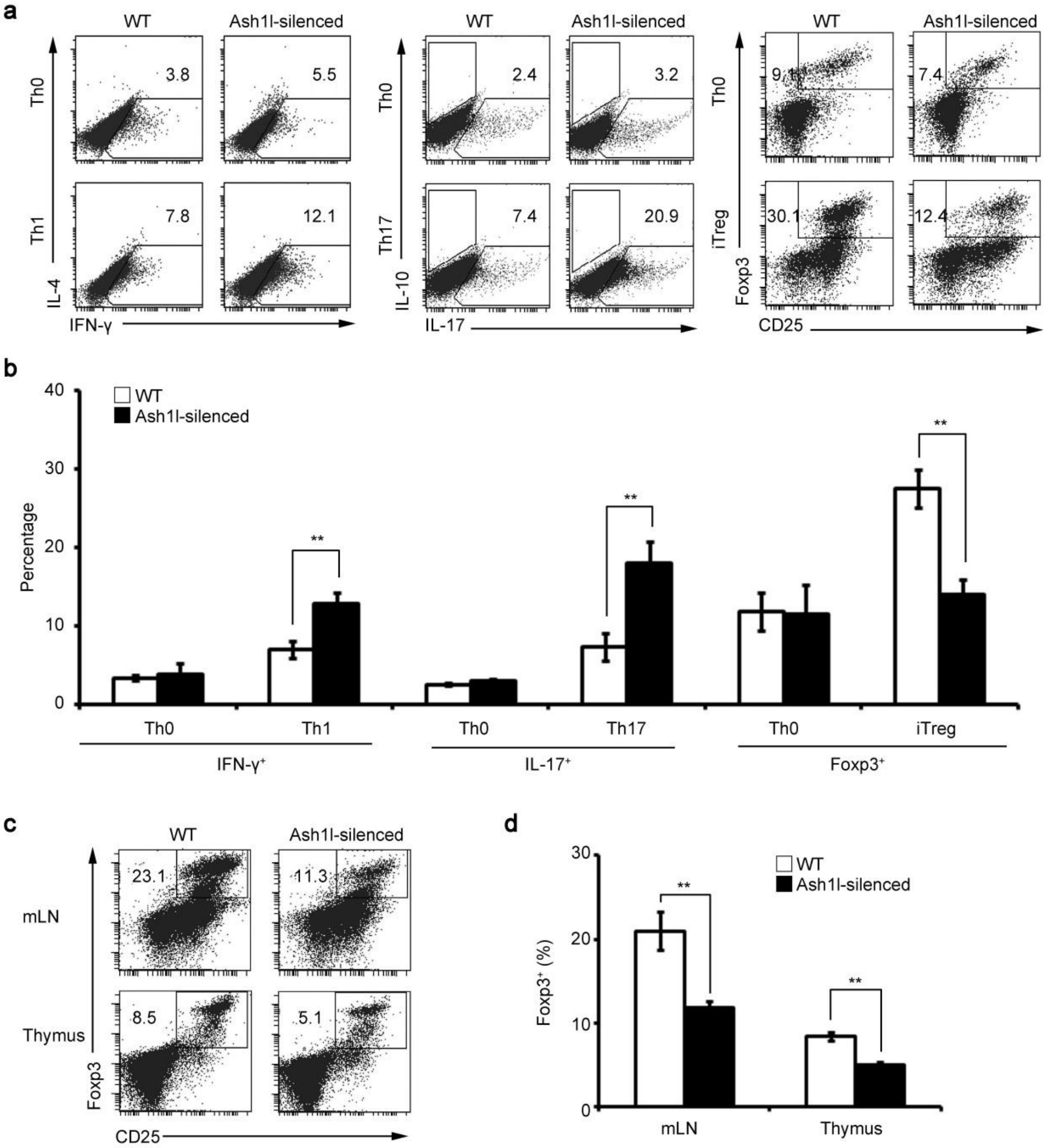

Supplementary Figure 2. Ash1l affects the induced differentiation of T cells under polarization conditions.

(**a, b**) Flow cytometry profiles the pooled spleens from WT and Ash1l-silenced mice (n = 3 mice per group), showing expression of IFN-γ, IL-17, and Foxp3 in the na ïve $CD4^+$ T cells (Th0) or in the $CD4^+$ T cells stimulated under indicated polarizing conditions (Th1, Th17 or iTreg cell skewing conditions respectively) for 3 days. Numbers beside outlined areas indicate percentage of cells in the $CD4^+$ gate.

(**c, d**) Flow cytometry profiles the mLN (mesenteric lymph nodes) and thymus from WT and Ash1l-silenced mice (n = 3 mice per group), showing expression of Foxp3 in the $CD4^+$ T cells stimulated under iTreg cell–skewing conditions (with TGF-β) for 3 days. Numbers beside outlined areas indicate percent $Foxp3^+$ cells in the $CD4^+$ gate. Error bars represent SD. Student's t test. **$p < 0.01$. Data are representative of three independent experiments (**a**, **c**) or are from three independent experiments (**b, d**; mean ± SD of technical triplicates).

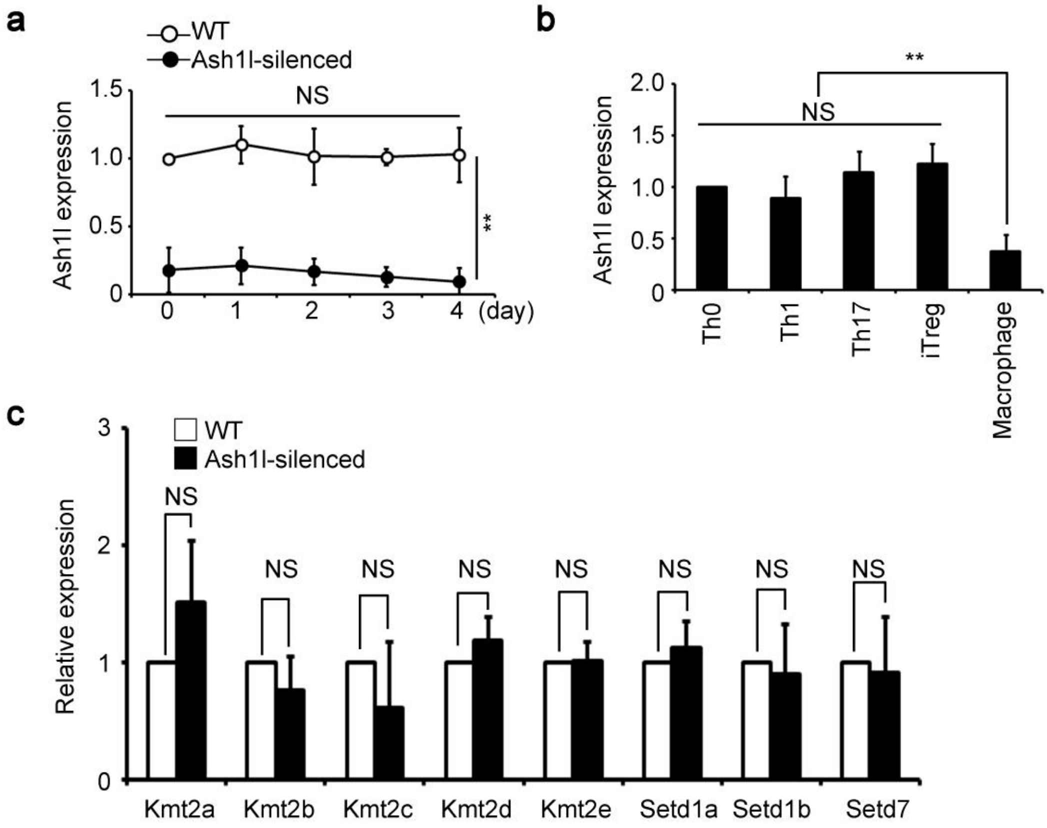

Supplementary Figure 3. Silencing of Ash1l does not affect the expression of other H3K4 methyltransferases.

(**a**) The mRNA level of Ash1l in the CD4$^+$ T cells stimulated under iTreg cell–skewing conditions (with TGF-β) for indicated times. Results are relative to the baseline Ash1l expression in unstimulated WT CD4$^+$ T cells, set as 1.

(**b**) The mRNA level of Ash1l in the na ïve CD4$^+$ T cells (Th0), T cells stimulated under indicated polarizing conditions (Th1, Th17 or iTreg cell skewing conditions respectively) for 3 days, and in macrophages. Results are relative to the baseline Ash1l expression in Th0 cells, set as 1.

(**c**) The mRNA levels of 8 H3K4 methyltransferases in WT and Ash1l-silenced na ïve CD4$^+$ T cells. Results are relative to those in WT na ïve CD4$^+$ T cells, set as 1.

Error bars represent SD. Student's t test. **$p < 0.01$. NS, not significant. All data are from three independent experiments (mean ± SD of technical triplicates).

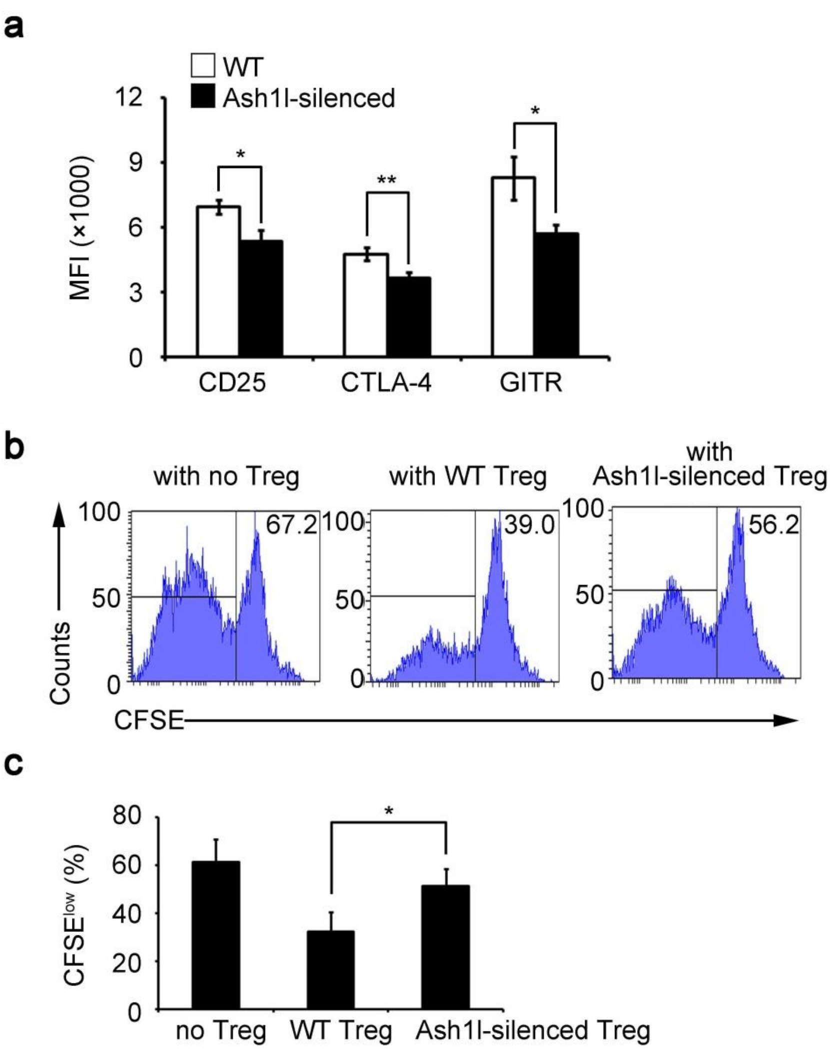

Supplementary Figure 4. Ash1l facilitates TGF-β-mediated Treg cell function.

(**a**) Flow cytometry profiles the expression of indicated costimulatory molecules on WT and Ash1l-silenced iTreg cells. Data are mean fluorescence intensity (MFI) ± SD, × 1000; n = 3.

(**b, c**) Proliferation of $CD45.1^+$ WT CFSE-labeled $CD4^+$ T cells incubated with BMDCs and WT or Ash1l-silenced iTreg cells, assessed by flow cytometry (**b**) and quantified (**c**) as dilution of CFSE at 72h. Numbers above plots indicates percent of $CFSE^{low}$ cells in the $CD45.1^+$ gate.

MFI, mean fluorescence intensity. Error bars represent SD. Student's t test. *p < 0.05, **p < 0.01. Data are from three independent experiments (**a, c**; mean ± SD of technical triplicates) or representative of three independent experiments (**b**).

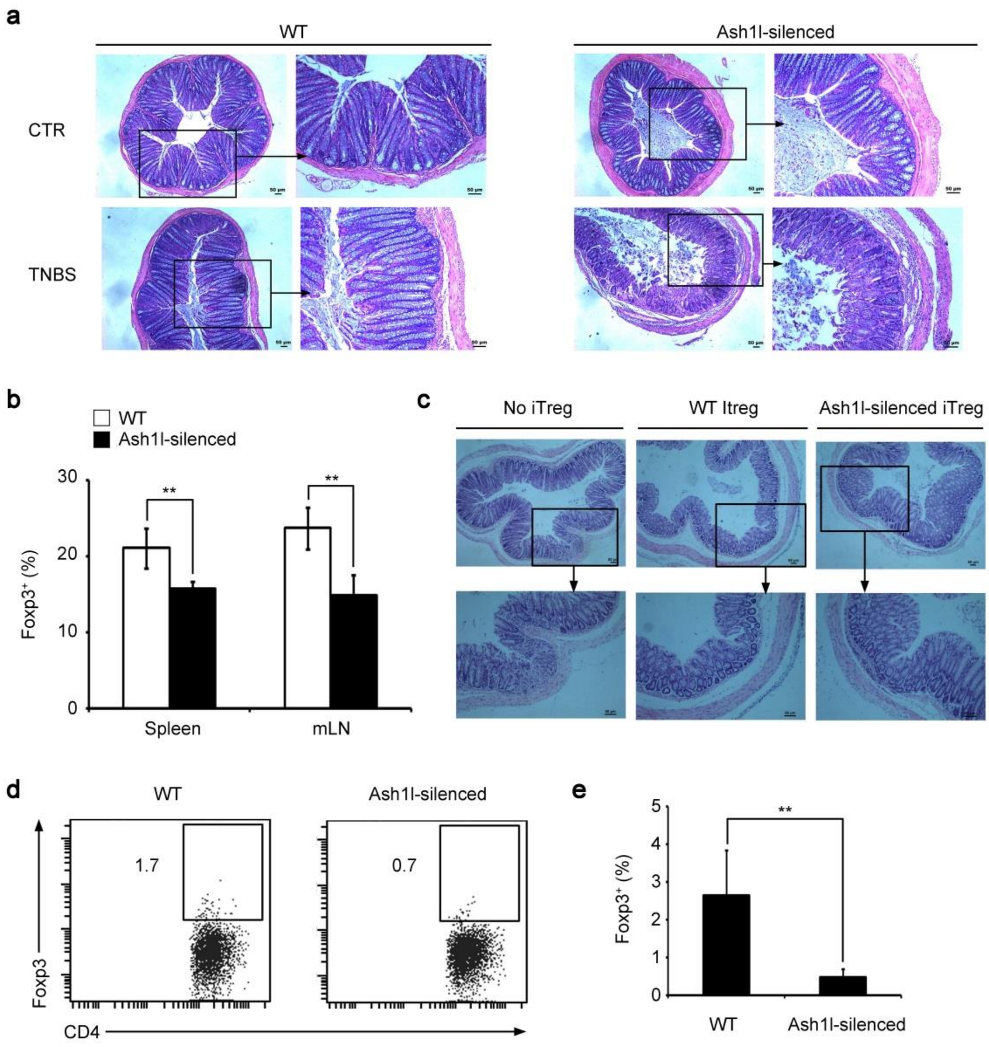

Supplementary Figure 5. *In vivo* silencing of Ash1l renders mice more susceptible to T cell-mediated colitis.

(**a**) TNBS-colitis was induced as in **Fig. 2a**. Histological sections of colons from WT and Ash1l-silenced mice (n = 5 mice per group) were examined at day3 after TNBS-induction. Outlined areas are shown at higher magnification at right. Scale bars represent 50μm.

(**b**) Percentages of Foxp3$^+$ Tregs in splenocytes and mLN (mesenteric lymph nodes) cells were determined at day3 after TNBS-induction.

(**c**) T cell contransfer colitis was induced as in **Fig. 2d**. Histological sections of colons from Rag1$^{-/-}$ mice transferred with WT CD4$^+$CD25$^-$ T cells with or without WT or Ash1l-silenced iTreg cells were examined at week 6 after T cell transfer. Outlined areas are shown at higher magnification at right. Scale bars represent 50μm.

(**d, e**) Representative flow cytometry (**d**) and quantification (**e**) of the percentages of Foxp3$^+$ Tregs in splenocytes of *Rag1*$^{-/-}$ mice 4 weeks following adoptive transfer of na ïve CD4$^+$ T cells from WT and Ash1l-silenced mice. Numbers indicate percentage of cells in the CD4$^+$ gate.

Error bars represent SD. Student's t test. **p < 0.01. Data are representative of three independent experiments (**a, c, d**) or from three independent experiments (**b, e**; mean ± SD of five mice).

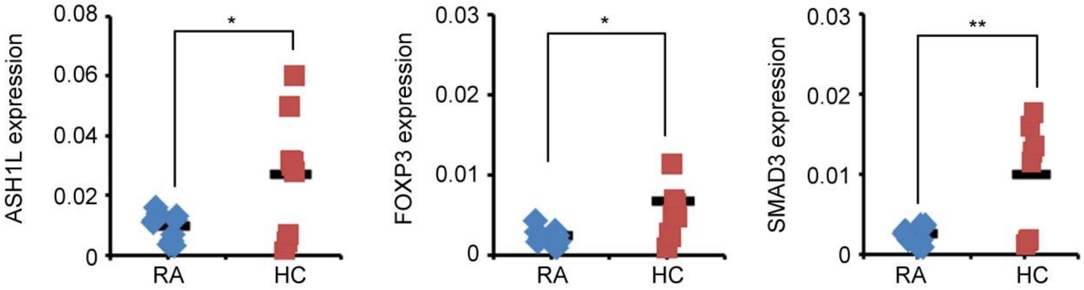

Supplementary Figure 6. ASH1L, FOXP3 and SMAD3 are significantly downregulated in CD4⁺ T cells from peripheral blood mononuclear cells (PBMCs) of patients with rheumatoid arthritis

mRNA expression of ASH1L, FOXP3 and SMAD3 in $CD4^+$ T cells sorted from the peripheral blood mononuclear cells (PBMCs) of 10 individuals with rheumatoid arthritis (RA) or 9 healthy controls (HC). Error bars represent SD. Student's t test. *p < 0.05, **p < 0.01. Data are shown as mean ± SD.

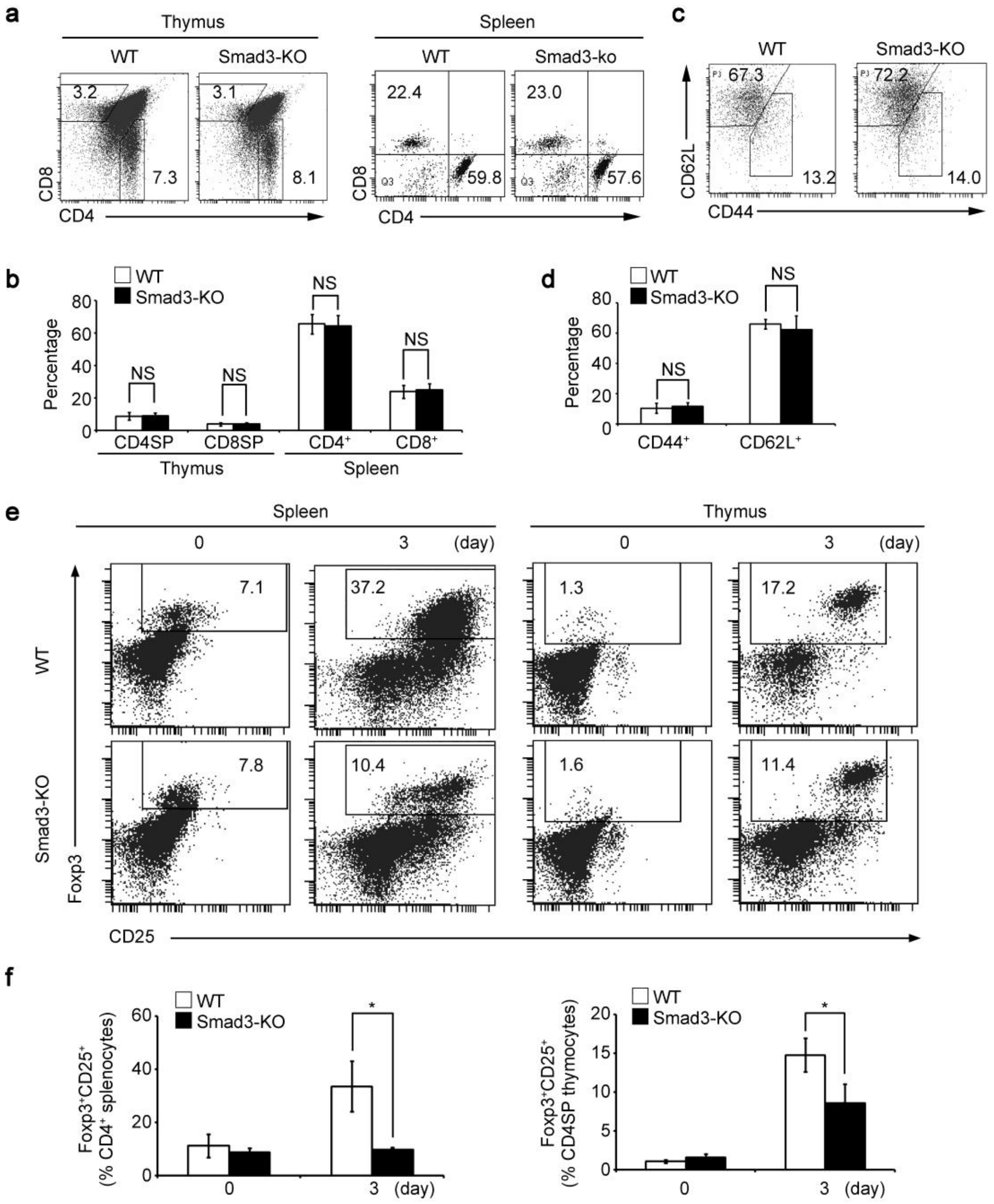

Supplementary Figure 7. Less iTreg cell generation in Smad3-KO mice.

(**a-d**) The proportion of CD4 and CD8 in the thymus and spleen (**a, b**), and the expression of CD44 and CD62L in peripheral $CD4^+$ T cells (**c, d**) from WT and Smad3-KO mice (n = 3 mice per group), assayed by flow cytometry (**a, c**) and quantified (**b, d**).

(**e, f**) The percentage of $Foxp3^+CD25^+$ in the $CD4^+$ T cells from WT and Smad3-KO splenocytes (left of e and f) or thymocytes (right of e and f) stimulated under iTreg cell–skewing conditions (with TGF-β) for 3 days, assayed by flow cytometry (**e**) and quantified (**f**).

Error bars represent SD. Student's t test. NS, not significant, *$p < 0.05$. Data are representative of three independent experiments (**a, c, e**) or from three independent experiments (**b, d, f**; mean ± SD of three mice).

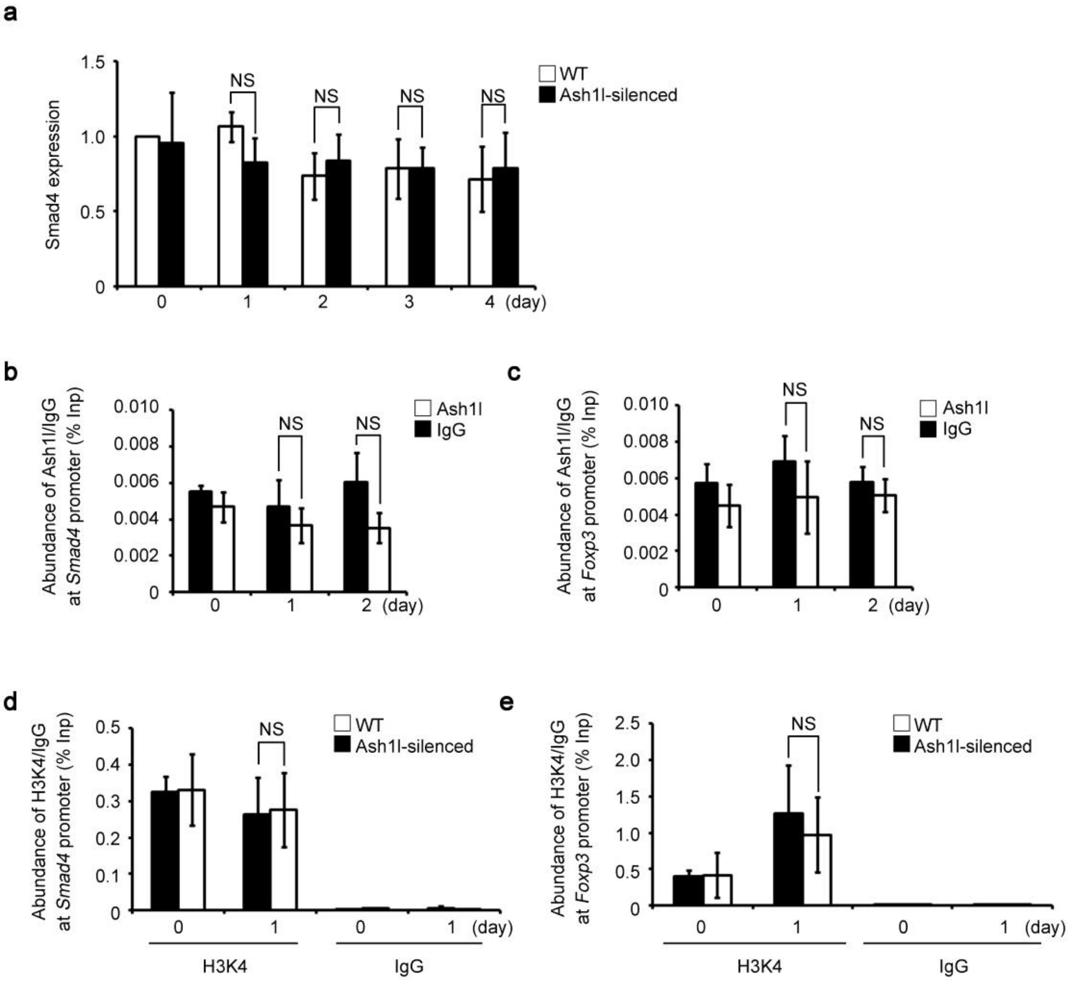

Supplementary Figure 8. Ash1l does not accumulate at *Smad4* or *Foxp3* promoter regions in response to TGF-β stimulation.

(**a**) The expression of Smad4 in WT and Ash1l-silenced $CD4^+$ T cells stimulated under iTreg cell–skewing conditions (with TGF-β) for indicated times, analyzed by quantitative PCR. Results are presented relative to the baseline expression in unstimulated WT $CD4^+$ T cells, set as 1.

(**b, c**) CHIP analysis of the recruitment of Ash1l to the *Smad4* (**b**) and *Foxp3* (**c**) promoter regions with anti-Ash1l antibody in $CD4^+$ T cells stimulated under iTreg cell–skewing conditions (with TGF-β) for indicated times. Normalized data are shown as percentage of input control (% Inp). IgG serves as a ChIP control.

(**d, e**) H3K4me3 modifications of the *Smad4* (**d**) and *Foxp3* (**e**) promoter regions. CHIP analysis of the trimethylation of histone H3 lysine 4 (H3K4me3) at the *Smad4* and *Foxp3* promoter regions in WT and Ash1l-silenced $CD4^+$ T cells stimulated under iTreg cell–skewing conditions (with TGF-β) for 1 day. Normalized data are shown as percentage of input control (% Inp). IgG serves as a ChIP control.

Error bars represent SD. Student's t test. NS, not significant. All data are from three independent experiments (mean ± SD of technical triplicates).

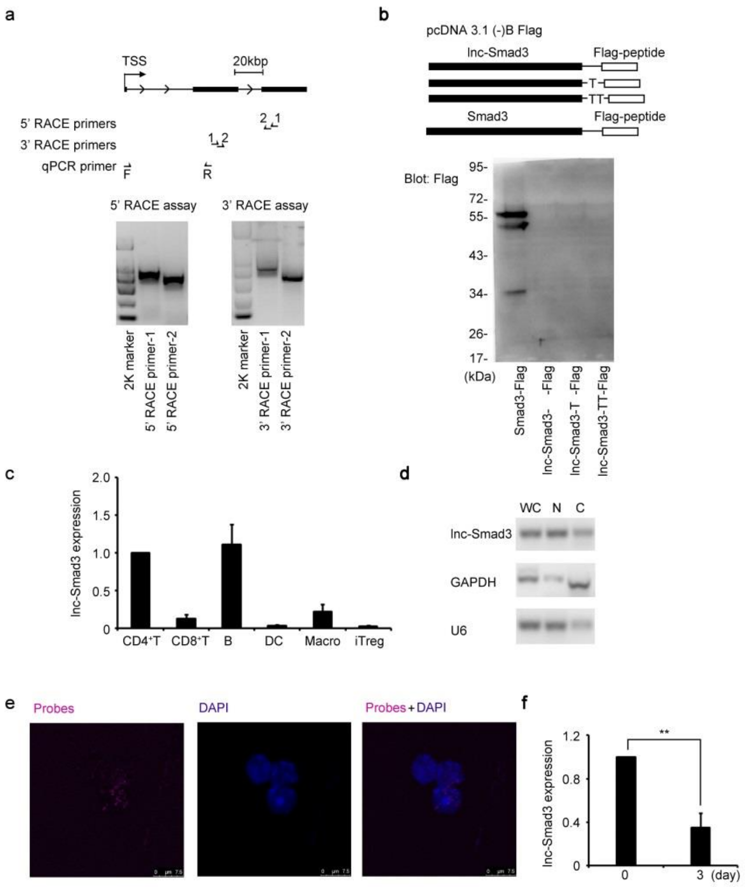

Supplementary Figure 9. Expression profiles of lnc-Smad3.

(**a**) TSS identified with 5' cap structures using two sets of 5'RACE primers and transcriptional ending site identified with 3' poly (A) using two sets of 3' RACE primers in RACE assay and illustration of the full-length lnc-Smad3 transcripts cloned from mouse B220^{+} B cells.

(**b**) Full-length lnc-Smad3 was cloned into the eukaryotic expression vector pcDNA3.1 with N-terminal start codon ATG and C-terminal Flag tag in all three coding patterns and these plasmids subsequently were transfected into HEK293T cells separately. After 48 hours, immunoblotting was used to detect the Flag-tagged protein. Smad3 with Flag tag severs as a positive control.

(**c**) Lnc-Smad3 expression in immune cells, including CD4^{+} T cells, CD8^{+} T cells, B cells, dendritic cells (DC), peritoneal macrophages and inducible Treg cells (iTreg). Results were relative to the mRNA amount of CD4^{+} T cells, set as 1.

(**d**) Semiquantitative PCR detection of Lnc-Smad3 in the whole cell extract (WC), nuclear (N) and cytoplasmic (C) fractions of CD4^{+} T cells. GAPDH and U6 serve as cytoplasmic and nuclear localization control, respectively.

(**e**) RNA FISH assay of lnc-Smad3 in naïve CD4^{+} T cells. Scale bars represent 7.5 μm. More than thirty cells have been examined and got similar results.

(**f**) Lnc-Smad3 expression in B cells stimulated with 10ng/ml TGF-β for 3 days. Results were relative to the baseline expression of lnc-Smad3 in unstimulated B cells, set as 1.

Error bars represent SD. Student's t test. **$p < 0.01$. Data are from three independent experiments (**c, f**; mean ± SD of technical triplicates) or representative of three independent experiments (**a**, **b**, **d**, **e**).

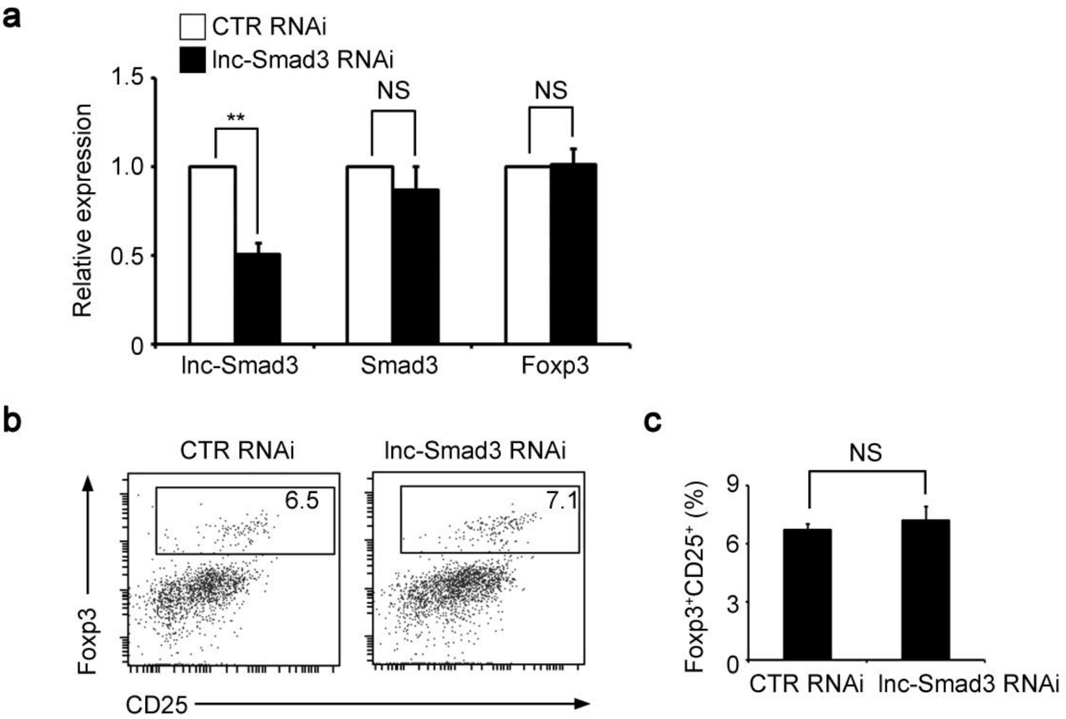

Supplementary Figure 10. Knockdown of lnc-Smad3 without TGF-β stimulation fails to induce iTreg cell generation.

(**a**) The expression of the lnc-Smad3, Smad3 and Foxp3 in CD4$^+$ T cells transduced and cultured with lentivirus-mediated Lnc-Smad3 RNAi or its control for 48 h. Results were relative to the expression of those in CD4$^+$ T cells transduced with control, respectively set as 1.

(**b, c**) Representative flow cytometry (**b**) and quantification (**c**) of the percentages of Foxp3$^+$ Tregs in CD4$^+$ T cells transduced and cultured as in **a**. Numbers in quadrants indicate percent cells in each.

Error bars represent SD. Student's t test. **p < 0.01. NS, not significant. Data are from three independent experiments (**a, c**; mean ± SD of technical triplicates) or representative of three independent experiments (**b**).

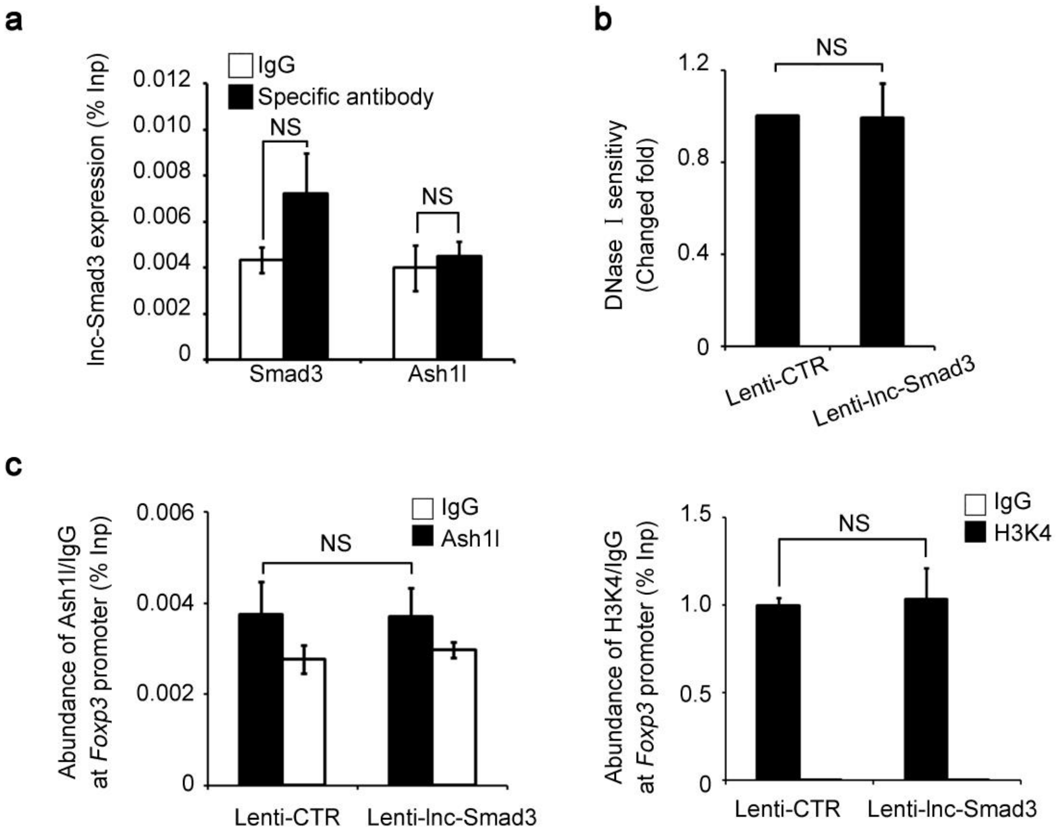

Supplementary Figure 11. Lnc-Smad3 does not regulate the chromatin state of *Foxp3* promoter region.

(**a**) Quantitative PCR detection of the lnc-Smad3 retrieved by Smad3 or Ash1l specific antibody compared with immunoglobulin G (IgG) in the RIP assay within $CD4^+$ T cells. Normalized data are shown as percentage of input control (% Inp). IgG serves as a RIP control.

(**b**) Chromatin accessibility of the *Foxp3* promoter region by quantitative PCR with DNase I pretreated nucleus of $CD4^+$ T cells transduced with a control lentivirus (Lenti-CTR) or lnc-Smad3-expressing lentivirus (Lenti-lnc-Smad3) and cultured under iTreg cell–skewing conditions (with TGF-β) for 2 days. Changed fold are concluded using $2^{\Delta Ct}$ with respect to $CD4^+$ T cells transduced with Lenti-CTR, set as 1.

(**c**) CHIP analysis of the accumulation of Ash1l and H3K4me3 modification at *Foxp3* promoter regions in $CD4^+$ T cells transduced and cultured as in **b**. Normalized data are shown as percentage of input control (% Inp). IgG serves as a ChIP control.

Error bars represent SD. Student's t test. NS, not significant. Data are from three independent experiments (mean ± SD of technical triplicates).

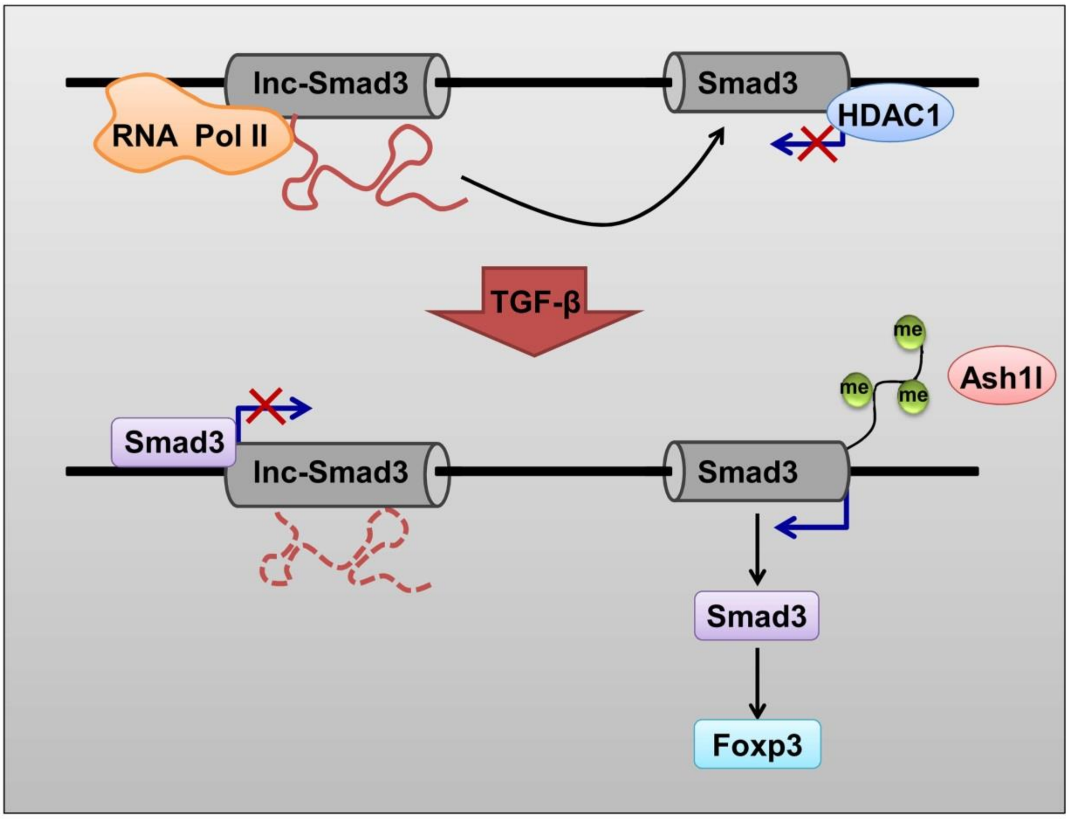

Supplementary Figure 12. Ash1l competes with lnc-Smad3 to promote iTreg cell polarization via upregulating TGF-β-mediated Smad3 expression. In naïve T cells, lnc-Smad3 turns off the transcription activation of the nearby gene locus *Smad3* by recruiting HDAC1. Upon TGF-β stimulation, activated Smad3 suppresses lnc-Smad3 expression by binding to its promoter region. TGF-β-mediated reduction of lnc-Smad3 relieves its suppression on *Smad3* promoter region, restoring the accessibility of *Smad3* promoter to Ash1l. Hence Ash1l is allowed to accumulate at *Smad3* promoter, increase the H3K4me3 modification and activate *Smad3* transcription. Consequently, Ash1l-upregulated Smad3 facilities Foxp3 expression as a transcription factor and finally enhances TGF-β-mediated Treg cell generation.

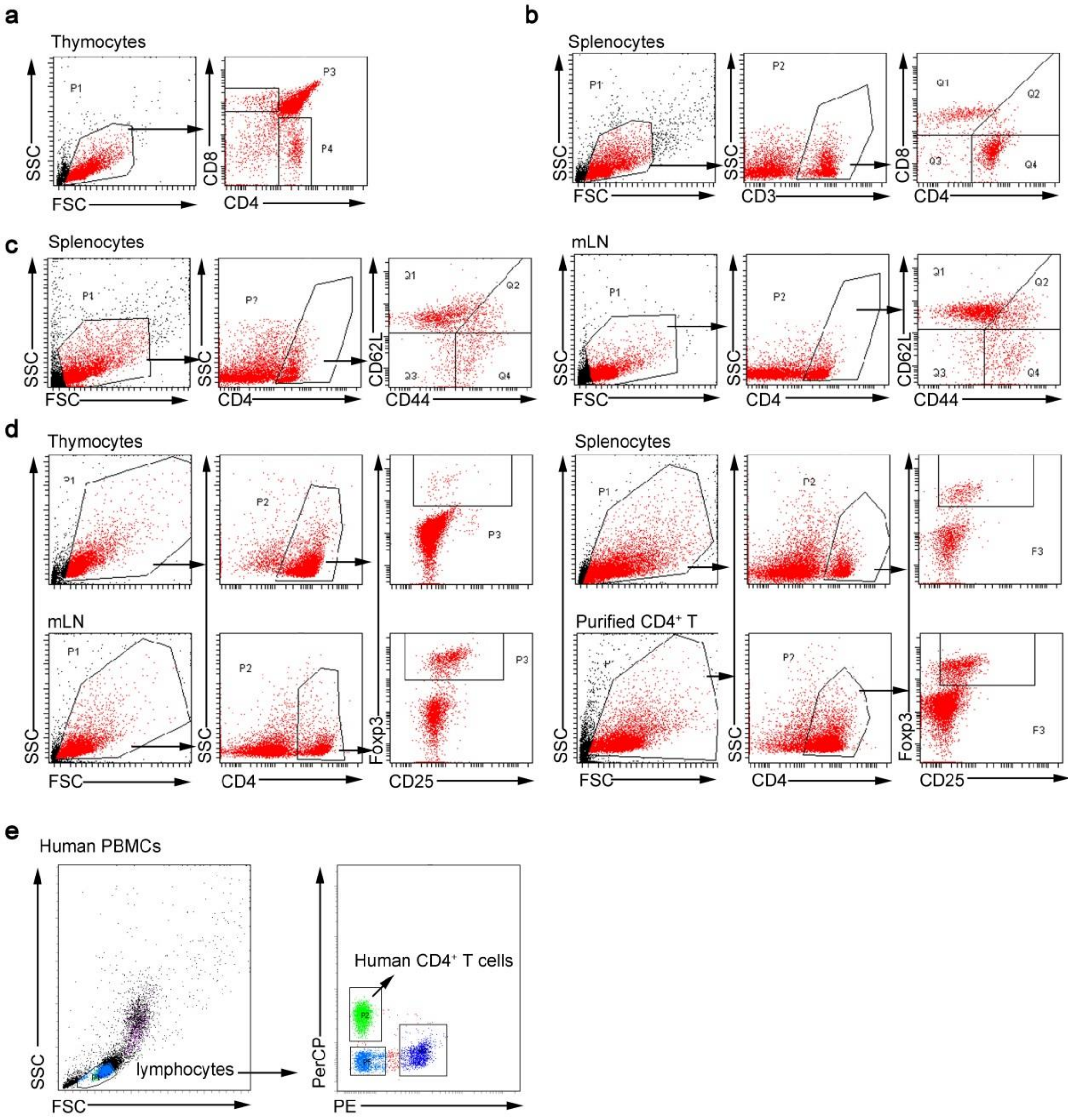

Supplementary Figure 13. The gating and sorting strategies of indicated cell subsets.

(**a, b**) The representative FACS plots show the gating strategies of $CD4^+$ and $CD8^+$ T cell subsets from the thymocytes or splenocytes of mice, corresponding to Supplementary Fig. 1a, b and Supplementary Fig. 7a, b.

(**c**) The representative FASC plots show the gating strategies of $CD62L^+$ and $CD44^+$ T cell subsets from splenocytes and mLN (mesenteric lymph nodes) of mice, corresponding to Supplementary Fig. 1c, d and Supplementary Fig. 7c, d.

(**d**) The representative FASC plots show the gating strategies of $Foxp3^+CD25^+$ T cell subset from splenocytes, mLN, thymocytes and purified $CD4^+$ T cells of mice, corresponding to Fig. 1a, b, d, e; Fig. 5g, h; Supplementary Fig. 1e, f ; Supplementary Fig. 2c, d; Supplementary Fig. 5d, e; Supplementary Fig. 7e, f and Supplementary Fig. 10b, c;

(**e**) The representative FASC plots show the sorting strategies of human $CD4^+$ T cell subset from human peripheral blood mononuclear cells (PBMCs), corresponding to Supplementary Fig. 6.

**Characteristic**	**RA (n=10)**	**HC (n=9)**
Male/Female, no.	4/6	4/5
Age, years, Median (range)	57±19	53±7
Disease duration, years, Median (range)	9.42 (0.2-30)	——
ESR, mm/hr, Median±SD	42.1±29.9	——
Presurgical CRP (mg/L), Median±SD	39.7±39	——
DAS28, Median±SD	5.7±1.7	——

Supplementary Table 1. Clinical characteristic of RA patients or healthy controls. Clinical features of the 10 RA patients and 9 healthy controls who contributed peripheral blood mononuclear cell (PBMC) samples. ESR: erythrocyte sedimentation rate. CRP: C-reactive protein. DAS28: Disease activity score.

Gene Symbol	Primers (5'-3')	
	Forward	Reverse
Smad2	AAGCCATCACCACTCAGAATTG	CACTGATCTACCGTATTTGCTGT
Smad3	CACGCAGAACGTGAACACC	GGCAGTAGATAACGTGAGGGA
Smad4	ACACCAACAAGTAACGATGCC	GCAAAGGTTTCACTTTCCCCA
Foxp3	CCCAGGAAAGACAGCAACCTT	TTCTCACAACCAGGCCACTTG
Ash1l	CGAAGGCACAGCCAGAG	GCAGGTAGGTCACGTCAA
lnc-Smad3	AGGCCAACGATCCAGGTTTA	ACATGTCTGGAGGCAATGGA
Kmt2a	GCAGATTGTAAGACGGCGAG	GAGAGGGGGTGTTCCTTCCTT
Kmt2b	CGTCACCACCCTTGACTCC	CATGTAGCTGGGACAAGAGGA
Kmt2c	TGTTCACAGTGTGGTCAATGTT	GAGGGTCTAGGCAGTAGGTATG
Kmt2d	GTGGCTGTTCCACACCCAG	AGCTTGAGCTTCTCAGCATCG
Kmt2e	AGATGCACTTACAATCAAGAGGG	AGGGCTGGTATAACCAATAGTC
Setd1a	TGCTGTCCCTCGTAGACTGG	GGCTCTTTCCGTTTTACCTTGA
Setd1b	TCCTCAAGCTCCGACAAGGAT	CGTCGATGTCTGAATCAATCTG
Setd7	GGACGATGACGGATTACCACA	ATTTCCCACGCCCATTCTTTT

Supplementary Table 2. Sequences of the primers for quantitative real time RT-PCR of mRNA levels.

Gene Symbol	Primers (5’-3’)	
	Forward	Reverse
Smad2	ACCACTGCGAGTAGTCTGAAAT	CGAGCCGTGGGTTCTTC
Smad3 (locus E)	AGCTGCGTGAAACGTAGACTTG	CCTGGATGGCTTTGGTGC
Smad3 (locus F)	GATCTCACCCAGCAGGAAA	GATTAGACTCACAGGGAACAGG
Smad3 (locus G)	TTTCGGAACTTCAGTAACACC	AAACGGGCCTCAAAGACA
Smad4	AGGAAGGGTGGGAACT	TCTTACCAAATGTGCC
Foxp3	ACCTTTTACCTCTGTGGTGAG	GTAGTTTTTTTTTCTTTGCTCTC
lnc-Smad3 (locus A)	GTTTACCTATGGCTTGACC	GATGAGGAACTGTGGGAG
lnc-Smad3 (locus B)	GAGTGGATCAAACAAGTT	AGTCTGGTAGTTCATTCA
lnc-Smad3 (locus C)	TCCGTGATATTGACTCTT	CATATTTTCTCAGGTGCT
lnc-Smad3 (locus D)	CAAAGGCTCTTGGTGATG	TCGTCTGACCCAGGCACTC

Supplementary Table 3. Sequences of the primers for amplification of promoter regions.
