# Supplementary material for: Ash1l and lnc-Smad3 coordinate Smad3 locus accessibility to modulate iTreg polarization and T cell autoimmunity
Source: Nat Commun. 2017 Jun 9;8:15818. doi: 10.1038/ncomms15818 (PMC5472765; doi:10.1038/ncomms15818)
Supplement: Peer Review File [file ncomms15818-s2.pdf]

Reviewers' comments:

Reviewer #1 (lncRNA, Th differentiation) (Remarks to the Author):

NCOMMS-16-29282

Title: Methyltransferase Ash1l controls autoimmunity by epigenetically upregulating Smad3 in iTreg cell polarization
Authors: Meng Xia, Juan Liu, Shuxun Liu, Kun Chen, Hongyu Lin, Minghong Jiang, Xiaoqing Xu, Yiquan Xue, Wei Liu, Yan Gu, Xiang Zhang, Zhiqing Li, Lin Yi, Youcun Qian, Chen Zhou, Ru Li, Xuan Zhang, Zhanguo Li, Xuetao Cao

Their model, which is well supported by their experimental approach and results, is that under basal conditions, a lncRNA, they call lnc-Smad3, inhibits Smad3 transcription via maintaining the promoter in a compact state, devoid of H3K4Me and H3K27Ac marks. Upon stimulation by TGF-b, activated suppresses lnc-Smad3 expression by binding to the lncRNA-Smad3 promoter. Loss of lnc-Smad3 relieves its suppression of the Smad3 promoter allowing recruitment of the histone methyltransferase, ASH1l, establishing H3K43Me marks. Increased expression of Smad3 induces increased expression of Foxp3 and enhances TGF-b induced Treg generation. They also look at a colitis model to show relevance in vivo and this seems satisfactory.

In general, experimental approach is pretty thorough. One might argue that conditional deletions would be preferable but the restoration experiments with the lentiviral vectors seem to get around that argument.
Overall, seems that this will be an important contribution to Treg biology, Smad biology, and lncRNA biology and expand our understanding of how lncRNAs function.

A couple of points that need to be tended to:
Supplementary Fig 1 has no stats, no indication of quantitation in multiple mice and seems necessary.
Fig 1 c & d, also no stats, quantitation in multiple animals, seems necessary
Figure 1 e, also no stats, quantitation in multiple animals, seems necessary
Supp Fig 2 no stats, no quantitation of multiple mice, seems necessary
Fig. 2-am inclined to give them a pass on histology as other supporting data have appropriate numbers and stats.

Reviewer #2 (T epigenetic) (Remarks to the Author):

The manuscript investigates the role of H3K4 methyltransferase Ash1l on TGF- β - induced Treg cell generation and the impact on T cell-mediated colitis in mice. The major findings of the study are (1) that Ash1l upregulates Smad3 expression by targeting the Smad3 promoter region (2) the discovery of a new lncRNA that silences Smad3 transcription. These findings indicate that Ash1l and lnc-Smad3 play opposing roles in the regulation of Smad3 expression. With TGF-β stimulation, Smad3 suppresses lnc-RNA transcription, thereby allowing Smad3 promoter accessibility to Ash1l.

In general, this is an important study in the field of Treg research and possibly, autoimmune disease, with a suggested impact factor of 3. The discovery of lnc-Smad3 is novel and relevant in that it opposes the role of Ash1l, also previously discovered by the present authors. In their previous paper, they found that Ash1l-silenced mice were more likely to have a bacterial infection and autoimmune

disease. In the present study, the authors have further explored the role of Ash1l in T cell immunology.

Recommendation:

Invite the authors to revise their manuscript to address specific concerns before a final decision is reached.

Major Comments:

Overall, the many experiments described in the manuscript were well-thought out and logically presented. The results section clearly described their approach in a serial manner, providing convincing evidence that at least in mice, these findings are clearly substantiated.

The statistical analysis was simple and appropriate, using only t-tests throughout. In addition, the experiments were described in sufficient detail to allow for subsequent replication by others. Finally, the discussion was based solely on the results, without overstating their findings.

However, some elements were lacking in the manuscript. The title perhaps overstated their findings. Specifically, using the phrase "controls autoimmunity" seems overreaching. In addition, the discovery of lnc-Smad3 as a novel and opposing epigenetic modifier to Ash1l should instead be included in the title.

Unfortunately, the hypothesis was not clearly stated. Rather, in the second paragraph of the introduction, it states "more specific chromatin-modifying enzymes still need to be identified". And, in paragraph four of the introduction, the authors state "…inspiring us to further explore the role of Ash1l in the regulatory control of T-cell-mediated immunity and homeostasis". It is difficult to disprove the null when there is no clearly stated hypothesis.

In addition, there was no discussion regarding the stability of the iTreg. In comparison to nTregs, iTregs are unstable, possibly resulting in a large population of ex-iTreg cells. Others have shown that Tregs can become unstable and lose FoxP3 expression in inflammatory conditions, then producing proinflammatory cytokines (eg. INF-g), causing tissue destruction. As a result, the use of iTregs may be of limited utility in immunotherapy. However, the authors state in the discussion that perhaps Ash1l can be a diagnostic marker or therapeutic target for an inflammatory disease, such as colitis. Finally, the limitations of the study were not mentioned in the discussion. In particular, the feasibility of using iTregs as a therapeutic intervention should have been discussed.

Minor comments:
Figures 1-3 were too small, whereas Figures 4-6 appropriately sized. Some of the Figures in 1-3 should be placed in the supplement and enlarge the remaining, as was done in subsequent figures.

On pg 18 it states "Recent research figures out that…" Entire manuscript should be written using formal language style.

Lack of line numbers throughout the manuscript.

Reviewer #3 (histone modification) (Remarks to the Author):

Xia et al. claim that the lysine methyltransferase (KMT) Ash1l enhances TGF-beta-mediated induction and activation of Transforming growth factor-β (TGF-β)-dependent regulatory T (iTreg cells). In vivo, they claim that Ash1l prevents the development of T cell-mediated colitis by enhancing Treg cell generation and functions. During this process, Ash1l promotes Foxp3 expression via up-regulation of Smad3 expression by directly methylating H3K4 on Smad3 promoter. They have identified a new long non-coding RNA (lncRNA), named lnc-Smad3, which they claim represses Smad3 expression in naïve CD4+ cells. Upon TGF-beta stimulation, Smad3 is overexpressed and inhibits lnc-Smad3 expression, allowing recruitment of Ash1l to Smad3 promoter.

While the findings are potentially interesting, in its current form the study fails to make a compelling case at present. Although many of the experiments are satisfactory, the most essential ones provide rather indirect evidences. The advance represented by the paper is not decisive enough. The work needs additional key data to consolidate the main conclusion. Moreover, some data seem over-interpreted and important points need to be clarified. For example, the conclusion that "lnc-Smad3 selectively suppresses Smad3 expression via closing the chromatin state of Smad3 promoter region, while TGF-β-induced Smad3 reduces lnc-Smad3 expression via accumulating at lnc-Smad3 promoter regions" is not based on experimental data. Thus, some modifications have to be put to improve the quality of the message given in the paper.

The following few points are intended as constructive suggestions to improve this work:

- Page 4: "Previous studies showed that Treg cells have a distinct histone H3 lysine 27 (H3K27) trimethylation (H3K27me3) landscape": please be more clear, distinct from what? Do you mean "specific"?

- Have the authors considered to check for the expression levels of the other members of H3K4 KMT family in Ash1l-silenced cells?
- Fig 1a, c, e: Statistics are missing. Specify the number of experiments in each figure panel (in all figures).

- Fig 3: Authors must try to express the full-length Ash1l in order to conclude. Indeed, the use of truncated forms of Ashl1 is not convincing at all. How can one conclude on the function of the full-length protein here?

- Fig 4: ChIP-qPCR results must be presented as % of input as in Fig 6a, not as (fold). Positive and negative controls must also be presented to validate Ash1l and H3K4me3 ChIP.

- Fig S4d: Authors must perform RNA-FISH to confirm the subcellular localization of lnc-Smad3. Indeed, it is also present in the cytoplasm this has to be clarified and/or commented at least.

- Fig 5d: Please detail how the results are normalized?

- Figure 6b and page 15: We cannot conclude any absence of interaction in the absence of a positive control! Please modify text accordingly and delete this panel of include it as a Supplemental figure.

- The main assessment that "lnc-Smad3 selectively suppresses Smad3 expression via closing the chromatin state of Smad3 promoter region, while TGF-β-induced Smad3 reduces lnc-Smad3 expression via accumulating at lnc-Smad3 promoter regions" is not proved. Authors must show a direct effect of lnc-Smad3 on Smad3 promoter to validate their conclusion.

- Figure legends and methods must be more detailed. Many important details are missing (especially statistics, number of experiments…). For example: how the RT-PCR results are normalized; how the

graphs on Fig 3 were built?

Minor comments

- Page 5: please replace “combining” by “combined”.

- Primer sequences must be given.

REVIEWERS' COMMENTS:

Reviewer #1 (Remarks to the Author):

The author's have adequately responded to reviewer concerns. Manuscript is an important contribution to Treg and lncRNA biology.

Reviewer #2 (Remarks to the Author):

Although a number of revisions were made to the original document based on reviewer's comments, please note further suggestions for improvement.

Reviewer 2 (Question 1): The title perhaps overstated their findings. Specifically, using the phrase “controls autoimmunity” seems overreaching. In addition, the discovery of lnc-Smad3 as a novel and opposing epigenetic modifier to Ash1l should instead be included in the title.
Author Response: According to the suggestion, we have changed our title as followed:
“Methyltransferase Ash1l controls T cell autoimmunity by iTreg cell polarization via suppression of lncRNA lnc-Smad3”
Reviewer Response: Incomplete. The phrase “controls autoimmunity” was changed to “controls T cell autoimmunity”. This still seems overreaching. “Is a mediator of T cell autoimmunity” is more appropriate. lnc-Smad3 was added as suggested.

Reviewer 2 (Question 2): The hypothesis was not clearly stated.
Author Response: We have added new contents in the Paragraph 3 and 4 of the Introduction section to raise our hypothesis more clearly and logically in the revised version as followed.
In Paragraph 3 on Page 4-5, “While these studies highlight the involvement of epigenetic modifications in Treg cell development, it still remains poorly understand how specific chromatin-modifying factors cooperate to regulate TGF-β-induced cellular signaling and eventually affect differentiation and maintenance of Foxp3+ Treg cells. Characterization of the detailed roles of novel epigenetic factors in this process is important for expanding the regulatory mechanism of immune tolerance and suggesting the potential interventions of inflammatory autoimmune disease.”
In Paragraph 4 on Page 5, “inspiring us to further explore the role of Ash1l in the control of T cell-mediated immunity and homeostasis”
Reviewer Response: Incomplete. Still, no clear hypothesis stated. “Exploring” role of Ash11 is not a hypothesis.

Reviewer 2 (Question 3 and 4): There was no discussion regarding the stability of the iTreg; And the limitations of the study were not mentioned in the discussion.
Author Response: According to the suggestion, we have added new contents in Paragraph 2 of the Discussion section on Page 18-19 about the limitation of our study in the revised version as followed.
“Our data show that Ash1l, Smad3 and Foxp3 are all downregulated in CD4+ T cells from PBMCs of patients with rheumatoid arthritis, which is in accordance with the reduced expression of Ash1l in colitis in the gene profiling data on line. Thus it will be intriguing to investigate whether the Ash1l/Smad3/Foxp3 pathway may be involved in iTreg cell development in human. We found that knockdown of Ash1l (at an interference efficiency of 40% with Ash1l specific siRNA) did not obviously affect Smad3 and Foxp3 expression with TGF-β stimulation in human CD4+ T cells (data not shown). Actually, though TGF-β could induce Foxp3 expression both in human and mouse CD4+ T cells, the induced human Foxp3+ cells show little in vitro suppressive activity and even secrete proinflammatory cytokines such as IFN-γ1. Thus whether the human Foxp3+ T cells induced by TGF-β stimulation in vitro could physiologically represent in vivo functional Treg cells are still controversial. The feasibility

of using iTreg cells as a therapeutic intervention is also limited by the stability of infused Treg cells in humans1. Together, it requires further investigation to clarify the role of Ash1l in human Treg cell development and human inflammatory immune diseases, especially via the improved gene manipulation systems and optimized methods for induction of immunosuppressive human iTreg cells."
Reviewer Response: Incomplete. The following sentences, "Thus whether the human Foxp3+ T cells induced by TGF-β stimulation in vitro could physiologically represent in vivo functional Treg cells are still controversial. The feasibility of using iTreg cells as a therapeutic intervention is also limited by the stability of infused Treg cells in humans," are the only limitations mentioned in the manuscript. Limitations still need more discussion. At least one separate paragraph should be devoted to limitations, rather than it being embedded in a paragraph of findings. Need comma after "Thus".

Question5: Figures 1-3 were too small, whereas Figures 4-6 appropriately sized. Some of the Figures in 1-3 should be placed in the supplement and enlarge the remaining, as was done in subsequent figures.
Response: We have moved previous Fig. 1c, d to new Supplementary Fig. 4a-c; previous Fig. 2d,h to new Supplementary Fig. 5a, c; previous Fig. 3e to new Supplementary Fig. 6 respectively in the revised version. We also reorganized the new Fig.1-3 to appropriate size.
Reviewer Response: Appropriate changes were made.

Question 6: On pg 18 it states "Recent research figures out that…" Entire manuscript should be written using formal language style.
Response: We have revised the sentence to "Rmrp, a lncRNA associated with human cartilage-hair hypoplasia, was shown to regulate the transcriptional expression of RORγt target genes, hence controlling the differentiation of Th17 cells and development of inflammatory diseases." on Page 20 in the revised version.
Reviewer Response: Appropriate changes were made.

Question7: Lack of line numbers throughout the manuscript.
Response: We have added line numbers throughout the manuscript in the revised version.
Reviewer Response: Appropriate changes were made.

Additional Reviewer comment: Line 405. Reword sentence – awkward – eg "located and regulates"

Reviewer #3 (Remarks to the Author):

The authors have performed an extensive revision to suitably address the original concerns.

Minor point: English writing and text can be substantially improved.

**Point-to-point responses**

**To Reviewer #1**

**Comments:** *Their model, which is well supported by their experimental approach and results, is that under basal conditions, a lncRNA, they call lnc-Smad3, inhibits Smad3 transcription via maintaining the promoter in a compact state, devoid of H3K4Me and H3K27Ac marks. Upon stimulation by TGF-b, activated suppresses lnc-Smad3 expression by binding to the lncRNA-Smad3 promoter. Loss of lnc-Smad3 relieves its suppression of the Smad3 promoter allowing recruitment of the histone methyltransferase, ASH1l, establishing H3K43Me marks. Increased expression of Smad3 induces increased expression of Foxp3 and enhances TGF-b induced Treg generation. They also look at a colitis model to show relevance in vivo and this seems satisfactory.*

*In general, experimental approach is pretty thorough. One might argue that conditional deletions would be preferable but the restoration experiments with the lentiviral vectors seem to get around that argument.*

*Overall, seems that this will be an important contribution to Treg biology, Smad biology, and lncRNA biology and expand our understanding of how lncRNAs function. A couple of points that need to be tended.*

**Response:** We deeply appreciate the positive comments and helpful advice. We have modified the manuscript accordingly with new data as described below.

***Question 1:*** *Supplementary Fig 1 has no stats, no indication of quantitation in multiple mice and seems necessary.*

**Response:** We have provided quantification results in new **Supplementary Fig. 1b, d, f** and **Supplementary Fig. 2b, d** (corresponding to previous **Supplementary Fig.1**) in the revised version.

***Question 2 and 3:*** *Fig 1 c & d, also no stats, quantitation in multiple animals, seems necessary. Figure 1 e, also no stats, quantitation in multiple animals, seems necessary*

**Response:** We have provided quantification results in new **Fig. 1b, e** and **Supplementary Fig. 4a, c** (corresponding to previous **Fig. 1a, e** and **Fig 1c, d** respectively) in the revised version.

***Question 3:*** *Supp Fig 2 no stats, no quantitation of multiple mice, seems necessary*

**Response:** We have provided quantification results in new **Supplementary Fig. 5b, e** (corresponding to previous **Supplementary Fig. 2**) in the revised version. Besides, we also provided quantification results in new **Supplementary Fig. 7b, d, f** (corresponding to previous **Supplementary Fig. 3a, b**) in the revised version.

***Question 4:*** *Fig. 2- I am inclined to give them a pass on histology as other supporting data have appropriate numbers and stats.*

**Response:** We have moved the histological results in previous **Fig. 2** into new **Supplementary Fig. 5a, c** in the revised version.

**To Reviewer #2**

**Comments:** *The manuscript investigates the role of H3K4 methyltransferase Ash1l on TGF- β - induced Treg cell generation and the impact on T cell-mediated colitis in mice. The major findings of the study are (1) that Ash1l upregulates Smad3 expression by targeting the Smad3 promoter region (2) the discovery of a new lncRNA that silences Smad3 transcription. These findings indicate that Ash1l and lnc-Smad3 play opposing roles in the regulation of Smad3 expression. With TGF-β stimulation, Smad3 suppresses lnc-RNA transcription, thereby allowing Smad3 promoter accessibility to Ash1l.*

*In general, this is an important study in the field of Treg research and possibly, autoimmune disease, with a suggested impact factor of 3. The discovery of lnc-Smad3 is novel and relevant in that it opposes the role of Ash1l, also previously discovered by the present authors. In their previous paper, they found that Ash1l-silenced mice were more likely to have a bacterial infection and autoimmune disease. In the present study, the authors have further explored the role of Ash1l in T cell immunology.*

*Recommendation:*

*Invite the authors to revise their manuscript to address specific concerns before a final decision is reached.*

*Major Comments:*

*Overall, the many experiments described in the manuscript were well-thought out and logically presented. The results section clearly described their approach in a serial manner, providing convincing evidence that at least in mice, these findings are clearly substantiated.*

*The statistical analysis was simple and appropriate, using only t-tests throughout. In addition, the experiments were described in sufficient detail to allow for subsequent replication by others. Finally, the discussion was based solely on the results, without overstating their findings.*

**Response:** We deeply appreciate the positive comments and insightful advice. Following the advice, we have modified the manuscript accordingly as described

below.

***Question 1:*** *The title perhaps overstated their findings. Specifically, using the phrase "controls autoimmunity" seems overreaching. In addition, the discovery of lnc-Smad3 as a novel and opposing epigenetic modifier to Ash1l should instead be included in the title.*

**Response:** According to the suggestion, we have changed our title as followed: "**Methyltransferase Ash1l controls T cell autoimmunity by iTreg cell polarization via suppression of lncRNA lnc-Smad3**"

***Question 2:*** *The hypothesis was not clearly stated.*

**Response:** We have added new contents in the **Paragraph 3** and **4** of the **Introduction section** to raise our hypothesis more clearly and logically in the revised version as followed.

In **Paragraph 3** on **Page 4-5**, "While these studies highlight the involvement of epigenetic modifications in Treg cell development, it still remains poorly understand how specific chromatin-modifying factors cooperate to regulate TGF-β-induced cellular signaling and eventually affect differentiation and maintenance of $Foxp3^+$ Treg cells. Characterization of the detailed roles of novel epigenetic factors in this process is important for expanding the regulatory mechanism of immune tolerance and suggesting the potential interventions of inflammatory autoimmune disease."

In **Paragraph 4** on **Page 5**, "inspiring us to further explore the role of Ash1l in the control of T cell-mediated immunity and homeostasis"

***Question 3 and 4:*** *There was no discussion regarding the stability of the iTreg; And the limitations of the study were not mentioned in the discussion.*

**Response:** According to the suggestion, we have added new contents in **Paragraph 2**

of the **Discussion section** on **Page 18-19** about the limitation of our study in the revised version as followed.

"Our data show that Ash1l, Smad3 and Foxp3 are all downregulated in CD4$^+$ T cells from PBMCs of patients with rheumatoid arthritis, which is in accordance with the reduced expression of Ash1l in colitis in the gene profiling data on line. Thus it will be intriguing to investigate whether the Ash1l/Smad3/Foxp3 pathway may be involved in iTreg cell development in human. We found that knockdown of Ash1l (at an interference efficiency of 40% with Ash1l specific siRNA) did not obviously affect Smad3 and Foxp3 expression with TGF-β stimulation in human CD4$^+$ T cells (data not shown). Actually, though TGF-β could induce Foxp3 expression both in human and mouse CD4$^+$ T cells, the induced human Foxp3$^+$ cells show little *in vitro* suppressive activity and even secrete proinflammatory cytokines such as IFN-γ[1]. Thus whether the human Foxp3$^+$ T cells induced by TGF-β stimulation *in vitro* could physiologically represent *in vivo* functional Treg cells are still controversial. The feasibility of using iTreg cells as a therapeutic intervention is also limited by the stability of infused Treg cells in humans[1]. Together, it requires further investigation to clarify the role of Ash1l in human Treg cell development and human inflammatory immune diseases, especially via the improved gene manipulation systems and optimized methods for induction of immunosuppressive human iTreg cells."

***Question5:*** *Figures 1-3 were too small, whereas Figures 4-6 appropriately sized. Some of the Figures in 1-3 should be placed in the supplement and enlarge the remaining, as was done in subsequent figures.*

**Response:** We have moved previous **Fig. 1c, d** to new **Supplementary Fig. 4a-c**;

previous **Fig. 2d,h** to new **Supplementary Fig. 5a, c**; previous **Fig. 3e** to new **Supplementary Fig. 6** respectively in the revised version. We also reorganized the new **Fig.1-3** to appropriate size.

***Question6:*** *On pg 18 it states "Recent research figures out that…" Entire manuscript should be written using formal language style.*

**Response:** We have revised the sentence to "Rmrp, a lncRNA associated with human cartilage-hair hypoplasia, was shown to regulate the transcriptional expression of RORγt target genes, hence controlling the differentiation of Th17 cells and development of inflammatory diseases." on **Page 20** in the revised version.

***Question7:*** *Lack of line numbers throughout the manuscript.*

**Response:** We have added line numbers throughout the manuscript in the revised version.

**To Reviewer #3**

**Comments:** *Xia et al. claim that the lysine methyltransferase (KMT) Ash1l enhances TGF-beta-mediated induction and activation of Transforming growth factor-β (TGF-β)-dependent regulatory T (iTreg cells). In vivo, they claim that Ash1l prevents the development of T cell-mediated colitis by enhancing Treg cell generation and functions. During this process, Ash1l promotes Foxp3 expression via up-regulation of Smad3 expression by directly methylating H3K4 on Smad3 promoter. They have identified a new long non-coding RNA (lncRNA), named lnc-Smad3, which they claim represses Smad3 expression in naïve CD4*$^{+}$ *cells. Upon TGF-beta stimulation, Smad3 is overexpressed and inhibits lnc-Smad3 expression, allowing recruitment of Ash1l to Smad3 promoter.*

*While the findings are potentially interesting, in its current form the study fails to make a compelling case at present. Although many of the experiments are satisfactory, the most essential ones provide rather indirect evidences. The advance represented by the paper is not decisive enough. The work needs additional key data to consolidate the main conclusion. Moreover, some data seem over-interpreted and important points need to be clarified. For example, the conclusion that "lnc-Smad3 selectively suppresses Smad3 expression via closing the chromatin state of Smad3 promoter region, while TGF-β-induced Smad3 reduces lnc-Smad3 expression via accumulating at lnc-Smad3 promoter regions" is not based on experimental data. Thus, some modifications have to be put to improve the quality of the message given in the paper.*

**Response:** We deeply appreciate the insightful comments and helpful advice. Following the advice, we have performed additional experiments to support our conclusions and modified the manuscript with new data accordingly as described below.

***Question 1:*** *- Page 4: "Previous studies showed that Treg cells have a distinct histone H3 lysine 27 (H3K27) trimethylation (H3K27me3) landscape": please be more clear, distinct from what? Do you mean "specific"?*

**Response:** In the revised version, we have corrected the description into "Previous studies showed that Treg cells have a distinct histone H3 lysine 27 (H3K27) trimethylation (H3K27me3) landscape from that of naïve T cells and other T help cells".

***Question2:*** *- Have the authors considered to check for the expression levels of the other members of H3K4 KMT family in Ash1l-silenced cells?*

**Response:** We have performed qRT-PCR assay to measure the expression levels of the other 8 H3K4 methyltransferases in WT and Ash1l-silenced naïve $CD4^+$ T cells. New data in **Supplementary Fig. 3c** showed that the deficiency of Ash1l does not affect the expression of other H3K4 methyltransferases. We have added new description on **Page 8** about this result.

***Question3:-*** *Fig 1a, c, e: Statistics are missing. Specify the number of experiments in each figure panel (in all figures).*

**Response:** We have provided quantification results in new **Fig. 1b, e** and **Supplementary Fig. 4a, c** (corresponding to previous **Fig. 1a, e** and **Fig 1c, d** respectively) in the revised version. We have added the description of the number of experiments or mice in all the figure legends in the revised version.

***Question4:-*** *Fig 3: Authors must try to express the full-length Ash1l in order to conclude. Indeed, the use of truncated forms of Ashl1 is not convincing at all. How can one conclude on the function of the full-length protein here?*

**Response:** We have indeed tried to express the full-length Ash1l, but failed in PCR amplification of the target Ash1l full sequence which is as long as 9kb. Actually, it's technically difficult to construct a vector or lentivirus carrying gene fragment of such length. And even if the expression vectors might be successfully constructed, their transfection and expression in cells would also be very hard. Therefore, we constructed three Ash1l truncations (fragmen1-3) as well as the point mutation Ash1llΔN (N2212I) in our researches. We do hope the reviewers and editor could

understand the technical limitations and difficulties regarding this issue. We have demonstrated in our previous paper that the Ash1l-fragment3 vector encoding 1,886–2,958aa with SET domain has H3K4 methyltransferase activity and the Ash1llΔN mutation lacks the H3K4 methyltransferase activity (Xia M, et al, *Immunity* 2013; **39**, 470-481)[2], which further validated the rationality and feasibility of our construction strategy.

***Question5:***- *Fig 4: ChIP-qPCR results must be presented as % of input as in Fig 6a, not as (fold). Positive and negative controls must also be presented to validate Ash1l and H3K4me3 ChIP.*

**Response:** We have presented our ChIP-qPCR results as % of input in new **Fig. 4a, b** and **Fig. 6c**. We have also added the results of IgG as negative control. Since Pol II is required for almost all expressed genes, we think ChIP experiment with Pol □ antibody could serve as the positive control to confirm the effectiveness of our ChIP assays.

***Question6:***- *Fig S4d: Authors must perform RNA-FISH to confirm the subcellular localization of lnc-Smad3. Indeed, it is also present in the cytoplasm this has to be clarified and/or commented at least.*

**Response:** We have performed the RNA-FISH experiment and presented the data in new **Supplementary Fig. 9e**. New data confirmed that lnc-Smad3 is largely located in the nucleus, in consistent with its function in regulating chromatin state of Smad3. However, a small portion of lnc-Smad3 is also detected in the cytoplasm. The biological role of cytoplasmic lnc-Smad3 awaits further investigations. We've added related description on **Page 13** and related discussion on **Page 20**.

***Question7:***- *Fig 5d: Please detail how the results are normalized?*

**Response:** The results in **Fig. 5d** are normalized to the lnc-Smad3 expression in unstimulated WT $CD4^+$ T cells, set as 1. We have added related description in the figure legend of **Fig. 5d** in the revised version,

***Question8:-*** *Figure 6b and page 15: We cannot conclude any absence of interaction in the absence of a positive control! Please modify text accordingly and delete this panel of include it as a Supplemental figure.*

**Response:** We have modified our conclusion to "However, we observed no obvious interactions either between lnc-Smad3 and Smad3 or between lnc-Smad3 and Ash1l" on **Page 15** in the revised version. We also moved previous **Fig. 6b** to new **Supplementary Fig. 11a**.

***Question9:-*** *The main assessment that "lnc-Smad3 selectively suppresses Smad3 expression via closing the chromatin state of Smad3 promoter region, while TGF-β-induced Smad3 reduces lnc-Smad3 expression via accumulating at lnc-Smad3 promoter regions" is not proved. Authors must show a direct effect of lnc-Smad3 on Smad3 promoter to validate their conclusion.*

**Response:** In our previous manuscript, we found that overexpression of lnc-Smad3 in $CD4^+$ T cells under TGF-β stimulation reduced the chromatin accessibility (**Fig. 6c**) and the binding of Ash1l (**Fig. 6d**), and the H3K4me3 modification at the *Smad3* promoter region (**Fig. 6d**), indicating that lnc-Smad3 selectively regulated the chromatin state of *Smad3*. To further validate our conclusion, in our revised version, we provided new data showing a direct suppressive effect of lnc-Smad3 on *Smad3* promoter via reporter assay (new **Fig. 6b**).

According to the reviewer's suggestion, we also investigated how lnc-Smad3 regulates the chromatin state of *Smad3* promoter region. It was reported in another study that histone deacetylase HDAC1, could bind to *Smad3* promoter region[3]. In our revised manuscript, we showed that HDAC1, accumulated at *Smad3* promoter in mouse naïve $CD4^+$ T cells (new **Fig. 7a, b**) and also interacted with lnc-Smad3 (new **Fig. 7c**). Lnc-Smad3 overexpression promoted the accumulation of HDAC1 to *Smad3* promoter without affecting HDAC1 expression (new **Fig. 7d, e**). These data suggest that lnc-Smad3 is involved in kept of the compact chromatin structure of the *Smad3* promoter by recruiting HDAC1. According to these new data, we have revised our

conclusion accordingly into “lnc-Smad3 recruits HDAC1 to *Smad3* promoter region and suppresses Smad3 transcription”

***Question10:-*** *Figure legends and methods must be more detailed. Many important details are missing (especially statistics, number of experiments…). For example: how the RT-PCR results are normalized; how the graphs on Fig 3 were built?*

**Response:** We have provided statistics and quantification of the flow cytometry data in **Fig. 1b, e**; **Supplementary Fig. 1b, d, f**; **Supplementary Fig. 2b, d** and **Supplementary Fig. 4a, c**. We have also revised the figure legends, including adding the number of experiments or mice and how the RT-PCR results are normalized in the revised version. On **Fig. 3a**, the mRNA expression of Smad2 is relative to that in unstimulated WT $CD4^+$ T cells, set as 1. On **Fig. 3c**, the mRNA expression of Smad2 is relative to that in WT $CD4^+$ T cells transduced with Lenti-CTR, set as 1.

***Question11:-*** *Page 5: please replace “combining” by “combined”.*

**Response:** We have replaced “combining” by “combined” in our revised version.

***Question12:-*** *Primer sequences must be given.*

**Response:** We have provided all the primer sequences in our revised version. The sequences of the primers for quantitative real time RT-PCR are shown in **Supplementary Table 2**. The primers for amplification of gene promoters are shown in **Methods** section for **Chromatin immunoprecipitation (CHIP) and chromatin accessibility analysis**.

**References:**

1. Sakaguchi, S., Miyara, M., Costantino, C.M. & Hafler, D.A. $FOXP3^+$ regulatory T cells in the human immune system. *Nat. Rev. Immunol.* **10**, 490-500 (2010).

2. Xia, M. *et al*. Histone methyltransferase ash1l suppresses interleukin-6 production and inflammatory autoimmune diseases by inducing the ubiquitin-editing enzyme A20. *Immunity* **39**, 470-481 (2013).

3. Tang, Y. N. *et al*. Epigenetic regulation of Smad2 and Smad3 by profilin-2 promotes lung cancer growth and metastasis. *Nat. Commun.* **6**, 8230 (2015).

**Point-to-point responses**

**To Reviewer #1**

**Comments:** *The authors have adequately responded to reviewer concerns. Manuscript is an important contribution to Treg and lncRNA biology.*

**Response:** We deeply appreciate the positive comments.

**To Reviewer #2**

**Comments:** *Although a number of revisions were made to the original document based on reviewer's comments, please note further suggestions for improvement.*

**Question 1:** *The title perhaps overstated their findings. Specifically, using the phrase "controls autoimmunity" seems overreaching. In addition, the discovery of lnc-Smad3 as a novel and opposing epigenetic modifier to Ash1l should instead be included in the title.*

*Author Response: According to the suggestion, we have changed our title as followed: "Methyltransferase Ash1l controls T cell autoimmunity by iTreg cell polarization via suppression of lncRNA lnc-Smad3"*

*Reviewer Response: Incomplete. The phrase "controls autoimmunity" was changed to "controls T cell autoimmunity". This still seems overreaching. "Is a mediator of T cell autoimmunity" is more appropriate. lnc-Smad3 was added as suggested.*

**Response:** We have carefully considered the suggestion. However, the phrase "is a mediator of T cell autoimmunity" may cause misunderstanding of the main finding of our work. We showed that Ash1l negatively regulates autoimmunity, however, the phrase "*Is a mediator of T cell autoimmunity*" may cause the confusion that Ash1l promotes autoimmunity. We think that the description "Ash1l controls T cell autoimmunity" has properly concluded our findings that deficiency of Ash1l renders mice more susceptible to the autoimmune disease due to T cell dysregulation. Thus, we think the title in the last version, "Methyltransferase Ash1l controls T cell autoimmunity by iTreg cell polarization via suppression of lncRNA lnc-Smad3"

would be better. Thanks for your consideration.

**Question 2:** *The hypothesis was not clearly stated.*

*Author Response: We have added new contents in the Paragraph 3 and 4 of the Introduction section to raise our hypothesis more clearly and logically in the revised version as followed.*

*In Paragraph 3 on Page 4-5, "While these studies highlight the involvement of epigenetic modifications in Treg cell development, it still remains poorly understand how specific chromatin-modifying factors cooperate to regulate TGF-β-induced cellular signaling and eventually affect differentiation and maintenance of Foxp3+ Treg cells. Characterization of the detailed roles of novel epigenetic factors in this process is important for expanding the regulatory mechanism of immune tolerance and suggesting the potential interventions of inflammatory autoimmune disease."*

*In Paragraph 4 on Page 5, "inspiring us to further explore the role of Ash1l in the control of T cell-mediated immunity and homeostasis"*

*Reviewer Response: Incomplete. Still, no clear hypothesis stated. "Exploring" role of Ash1l is not a hypothesis.*

**Response:** We have clarified our hypothesis in the revised Introduction section as followed.

*In Paragraph 2 on page 4*, "So we suspect that epigenetic modifiers or enzymes may play potential roles in regulating TGF-β-induced cellular signalling and affect differentiation and maintenance of $Foxp3^+$ Treg cells."

*In Paragraph 4 on page 5*, "Combined, these data indicate a potential connection between Ash1l and T cell-mediated autoimmune disease."

**Question 3 and 4:** *There was no discussion regarding the stability of the iTreg; And the limitations of the study were not mentioned in the discussion.*

*Author Response: According to the suggestion, we have added new contents in Paragraph 2 of the Discussion section on Page 18-19 about the limitation of our study in the revised version as followed.*

*"Our data show that Ash1l, Smad3 and Foxp3 are all downregulated in CD4+ T cells from PBMCs of patients with rheumatoid arthritis, which is in accordance with the reduced expression of Ash1l in colitis in the gene profiling data on line. Thus it will be intriguing to investigate whether the Ash1l/Smad3/Foxp3 pathway may be involved in iTreg cell development in human. We found that knockdown of Ash1l (at an interference efficiency of 40% with Ash1l specific siRNA) did not obviously affect Smad3 and Foxp3 expression with TGF-β stimulation in human CD4+ T cells (data not shown). Actually, though TGF-β could induce Foxp3 expression both in human and mouse CD4+ T cells, the induced human Foxp3+ cells show little in vitro suppressive activity and even secrete proinflammatory cytokines such as IFN-γ1. Thus whether the human Foxp3+ T cells induced by TGF-β stimulation in vitro could physiologically represent in vivo functional Treg cells are still controversial. The feasibility of using iTreg cells as a therapeutic intervention is also limited by the stability of infused Treg cells in humans1. Together, it requires further investigation to clarify the role of Ash1l in human Treg cell development and human inflammatory immune diseases, especially via the improved gene manipulation systems and optimized methods for induction of immunosuppressive human iTreg cells."*

*Reviewer Response: Incomplete. The following sentences, "Thus whether the human Foxp3+ T cells induced by TGF-β stimulation in vitro could physiologically represent in vivo functional Treg cells are still controversial. The feasibility of using iTreg cells as a therapeutic intervention is also limited by the stability of infused Treg cells in humans," are the only limitations mentioned in the manuscript. Limitations still need more discussion. At least one separate paragraph should be devoted to limitations, rather than it being embedded in a paragraph of findings. Need comma after "Thus".*

**Response:** We have discussed in detail the limitation of iTreg cell generation and application in human as followed.

*In new added Paragraph 2 of Discussion section on Page 18-19*, "Notably, whether the human $Foxp3^+$ T cells induced by TGF-β stimulation *in vitro* could physiologically represent *in vivo* functional Treg cells are still controversial. Though TGF-β could induce Foxp3 expression both in human and mouse $CD4^+$ T cells, the

induced human Foxp3$^+$ cells are functionally heterogeneous, with nonregulatory subpopulations showing little *in vitro* suppressive activity and even secrete proinflammatory cytokines such as IFN-γ[27]. Highly specific Treg cell surface markers besides Foxp3 are required to identify and purify the regulatory subpopulations. The feasibility of using iTreg cells as a therapeutic intervention in human diseases is also limited by the stability of infused Treg cells. Considering the possible plasticity of Treg cells towards other pathogenic T cell subsets such as Th17 cells, it is necessary to confirm the purity and stability of Treg cells during Treg cell-based therapy in humans. Besides, the detailed molecular mechanisms of Treg cell-mediated suppression in humans still remain to be determined. Together, it requires further investigation to clarify the role of Ash1l in human Treg cell development and human inflammatory immune diseases, especially via the improved methods for induction of immunosuppressive, purified and stable human iTreg cells."

**Question5:** *Figures 1-3 were too small, whereas Figures 4-6 appropriately sized. Some of the Figures in 1-3 should be placed in the supplement and enlarge the remaining, as was done in subsequent figures.*

*Response: We have moved previous Fig. 1c, d to new Supplementary Fig. 4a-c; previous Fig. 2d,h to new Supplementary Fig. 5a, c; previous Fig. 3e to new Supplementary Fig. 6 respectively in the revised version. We also reorganized the new Fig.1-3 to appropriate size.*

*Reviewer Response: Appropriate changes were made.*

**Response:** We deeply appreciate the positive comments.

**Question 6:** *On pg 18 it states "Recent research figures out that…" Entire manuscript should be written using formal language style.*

*Response: We have revised the sentence to "Rmrp, a lncRNA associated with human cartilage-hair hypoplasia, was shown to regulate the transcriptional expression of RORγt target genes, hence controlling the differentiation of Th17 cells and development of inflammatory diseases." on Page 20 in the revised version.*

*Reviewer Response: Appropriate changes were made.*

**Response:** We deeply appreciate the positive comments.

**Question7:** *Lack of line numbers throughout the manuscript.*

*Response: We have added line numbers throughout the manuscript in the revised version.*

*Reviewer Response: Appropriate changes were made.*

**Response:** We deeply appreciate the positive comments.

**Additional Reviewer comment:** *Line 405. Reword sentence – awkward – eg "located and regulates"*

**Response:** We have corrected the sentence into "Lnc-Smad3 regulates the chromatin state of *Smad3* promoter mainly in the nucleus".

**To Reviewer #3**

**Comments:** *The authors have performed an extensive revision to suitably address the original concerns.*

*Minor point: English writing and text can be substantially improved.*

**Response:** We deeply appreciate the positive comments and helpful advice. We have modified the manuscript under the help of native English speakers.
